# Unraveling the protein kinase C/NDRG1 signaling network in breast cancer

**DOI:** 10.1186/s13578-024-01336-z

**Published:** 2024-12-30

**Authors:** C. Saponaro, M. Damato, E. Stanca, S. Aboulouard, F. A. Zito, S. De Summa, D. Traversa, L. Schirosi, S. Bravaccini, F. Pirini, E. Fonzi, M. Tebaldi, M. Puccetti, A. Gaballo, L. Pantalone, M. Ronci, L. Magnani, D. Sergi, A. Tinelli, S. Tacconi, L. Siculella, A. M. Giudetti, I. Fournier, M. Salzet, M. Trerotola, D. Vergara

**Affiliations:** 1 Pathology Department, IRCCS Istituto Tumori “Giovanni Paolo II”, 70124 Bari, Italy; 2 Department of Experimental Medicine, University of Salento, Lecce, Italy; 3 Laboratoire Protéomique, Réponse Inflammatoire et Spectrométrie de Masse (PRISM), Lille University, Inserm, CHU Lille, U1192 Lille, France; 4 Molecular Diagnostics and Pharmacogenetics Unit, IRCCS Istituto Tumori “Giovanni Paolo II”, 70124 Bari, Italy; 5 Department of Medicine and Surgery, University of Enna “Kore”, 94100 Enna, Italy; 6 Biosciences Laboratory, IRCCS Istituto Romagnolo per lo Studio dei Tumori (IRST) “Dino Amadori”, Meldola, Italy; 7 Unit of Biostatistics and Clinical Trials, IRCCS Istituto Romagnolo per lo Studio dei Tumori (IRST) “Dino Amadori”, Meldola, Italy; 8 Azienda Unità Sanitaria Locale di Imola, Imola, Italy; 9 CNR Nanotec, Institute of Nanotechnology, Via Monteroni, 73100 Lecce, Italy; 10 Laboratory of Cancer Pathology, Center for Advanced Studies and Technology (CAST), “G. d’Annunzio” University of Chieti-Pescara, Chieti, Italy; 11 Department of Medical, Oral and Biotechnological Sciences, “G. d’Annunzio” University of Chieti-Pescara, Chieti, Italy; 12 The Breast Cancer Now Toby Robins Research Centre, The Institute of Cancer Research, London, UK; 13 Department of Surgery and Cancer, Imperial College London, London, UK; 14 Department of Oncology and Haemato-Oncology, Università Degli Studi di Milano, Milan, Italy; 15 Department of Radiology, V. Fazzi Hospital, 73100 Lecce, Italy; 16 Department of Obstetrics and Gynecology and CERICSAL, (CEntro di RIcerca Clinico SALentino), “Veris Delli Ponti Hospital”, 73020 ScorranoScorrano (Lecce), Italy; 17 Department of Biology and Biotechnology “Charles Darwin”, Sapienza University of Rome, P.Le Aldo Moro 5, 00185 Rome, Italy; 18 Department of Biological and Environmental Sciences and Technologies (DiSTeBA), University of Salento, Lecce, Italy

**Keywords:** NDRG1, Breast cancer, TNBC, PKC

## Abstract

N-myc downstream-regulated gene 1 (NDRG1) is a member of the NDRG family of intracellular proteins and plays a central role in a wide range of biological processes including stress response, differentiation, and metabolism. The overexpression of NDRG1 is an indicator of poor prognosis in various types of cancer. Here, we found that NDRG1 is an independent prognostic marker of poor outcome in breast cancer (BC). Analysis of the TCGA dataset showed a significant positive correlation between *NDRG1* and *PRKCA* expression, suggesting a mechanistic role of protein kinase C (PKC) in the regulation of NDRG1. We then assessed the hypothesis that PKC might modulate the activity of NDRG1, and observed that different acute stress conditions converging on PKC activation lead to enhanced NDRG1 expression. This mechanism was found to be specific for NDRG1 as the expression of other NDRG members was not affected. Moreover, CRISPR-based inhibition of NDRG1 expression was obtained in a BC cell line, and showed that this protein is a key driver of BC cell invasion through the Rho-associated coiled-coil containing protein kinase 1 (ROCK1)/phosphorylated cofilin pathway that regulates stress fiber assembly, and the modulation of extracellular matrix reorganization related genes. Together, our findings highlight the potential of NDRG1 as a new BC biomarker and uncover a novel mechanism of regulation of NDRG1 expression that might lead to innovative therapeutic strategies.

**Supplementary Information:**

The online version contains supplementary material available at 10.1186/s13578-024-01336-z.

## Introduction

N-myc downstream-regulated gene 1 (NDRG1, also known as CAP43, DRG1, RTP) plays a central role in various biological processes including invasion, differentiation, and metabolism [1–3]. NDRG1 is part of the NDRG family that includes four members, namely, NDRG-1, -2, -3, and -4, that share approximately 57–65% homology at the amino acid level [4]. NDRG1 is ubiquitously expressed in a wide range of tissues and localizes to different cellular compartments including cytosol and microtubules (https://www.proteinatlas.org/ENSG00000104419-NDRG1/subcellular). DNA damage induces a redistribution of NDRG1 to the nucleus [5]. The truncation of NDRG1 near the N-terminus domain and the phosphorylation at the serine 330 may affect its ability to translocate to the nucleus [6]. The transcriptional regulation of NDRG1 was initially investigated in N-Myc knockout mouse embryos [7] and further demonstrated in vitro [8]. These studies revealed that NDRG1 is repressed by Myc on the core promoter region. In addition to being regulated by c-Myc, the expression of NDRG1 is controlled by the transcription factor HIF-1 (hypoxia-inducible factor 1), the T-box transcription factor TBX2, and p53 [9–11]. Several studies have begun to elucidate the correlation between NDRG1 expression and clinical features. The overexpression of NDRG1 is an indicator of poor prognosis in various tumor types including hepatocellular carcinoma, non–small cell lung cancer (NSCLC), and breast cancer (BC) [12–14]. In other cancer types, such as colorectal cancer, pancreatic cancer, and esophageal squamous cell carcinoma, low NDRG1 expression was significantly associated with worse overall survival [15, 16], suggesting tissue-specific functions of NDRG1. In BC, NDRG1 regulates lipid metabolism and vesicle transport. In detail, silencing of NDRG1 increased the fatty acid incorporation into neutral lipids and lipid droplets [2]. NDRG1 also regulates the expression of proteins involved in the regulation of the endoplasmic reticulum-to-endosome axis [17]. Moreover, NDRG1 belongs to a set of vascular endothelial growth factor (VEGF) genes that are correlated with distant metastases [18].

In light of this scenario, knowledge of the pro-metastatic signaling of NDRG1 appears urgently needed to provide novel insights on the molecular mechanisms that drive metastatic dissemination and to design next-generation targeted therapeutic strategies. Here, we investigated the prognostic potential of NDRG1 expression in BC and dissected the mechanisms by which activated PKC modulates NDRG1 expression.

## Materials and methods

### Case series

Twelve BC patients (6 Triple Negative, 3 Luminal A, and 3 Luminal B/Her2 positive) were enrolled at the Hospital Santa Maria della Scaletta, Imola (Italy). The study was conducted in accordance with ethical standards, the Declaration of Helsinki, and national and international guidelines, and was approved by local ethics committee (CE AVEC-protocol number 10547). All of the patients enrolled in the study have signed an informed consent for the use of the results for research purposes. Two hundred and eleven retrospective, non-consecutive primary invasive BC samples were collected at the Istituto Tumori “Giovanni Paolo II” of Bari (Italy). The study was approved by the Ethics Committee of the Istituto Tumori “Giovanni Paolo II” (no. 1310/CE of July 2023). Table S1 summarizes the clinicopathological characteristics of the entire cohort.

### Immunohistochemistry

Consecutive sections of 4-µm thickness were cut from formalin-fixed paraffin-embedded (FFPE) samples and stained with an indirect immunoperoxidase method using the BenchMark XT automated staining instrument (Ventana Medical Systems) and analysed as described in the Supplemental Experimental procedures.

### Mass spectrometry analysis and database searching

The mass spectrometry analysis on the peptides was gained in reverse phase, using a chromatography system equipped with a pre-column (Acclaim PepMap 75 μm ID × 2 cm, 3 µm, Thermo Scientific) to pre-concentrate the peptides, and an analytical column (Acclaim PepMap RSLC 75 µm ID × 50 cm, 2 µm, Thermo Scientific), used for their separation. Elution was carried out using a 2 h gradient of ACN/0.1% TFA starting from 5 to 30% for 120 min at a flow rate of 300 nL/min. The chromatographic system was coupled with a Q-Exactive Orbitrap mass spectrometer (Thermo Scientific) containing a nano-electrospray ionization source.

### Knockout of NDRG1 by the CRISPR/Cas9 approach

The sequences of the sgRNAs were as follows: NDRG1 CRISPR #1: 5′-GTTCATGCCGATGTCATGGT-3′ (strand antisense); NDRG1 CRISPR #2: 5′-GCAGGATGTAGACCTCGCTG-3′ (strand sense). These sgRNAs were cloned into the plasmid LentiCRISPRv2 (Addgene #52,961). Lentiviruses were collected from culture supernatant of HEK293T cells at 48 h after co-transfection with LentiCRISPRv2-sgRNA and with the packaging plasmids pVSVg (Addgene #8454) and psPAX2 (Addgene #12,260). The lentiviral particles were used to infect the target cells. Stable functional knock-out of *NDRG1* was obtained by selection in puromycin (3 µg/mL). Western blotting analysis of cell lysates was performed after two weeks of selection and revealed successful inhibition of the *NDRG1* expression by the NDRG1 CRISPR #2 sgRNA.

### Western blotting analysis

Cell lysates were extracted in RIPA buffer (Cell Signaling) and quantified by the BRADFORD method (Bio-RAD). Twenty-five μg of proteins were mixed 1:1 with Laemmli buffer (Sigma) boiled for 5 min, separated by 12% SDS-PAGE, and transferred to the Hybond ECL nitrocellulose membrane (GE Healthcare) and immunoblotted as described in the Supplemental Experimental procedures.

## Results

### NDRG1 is highly expressed in TNBCs

**Fig. 1:**
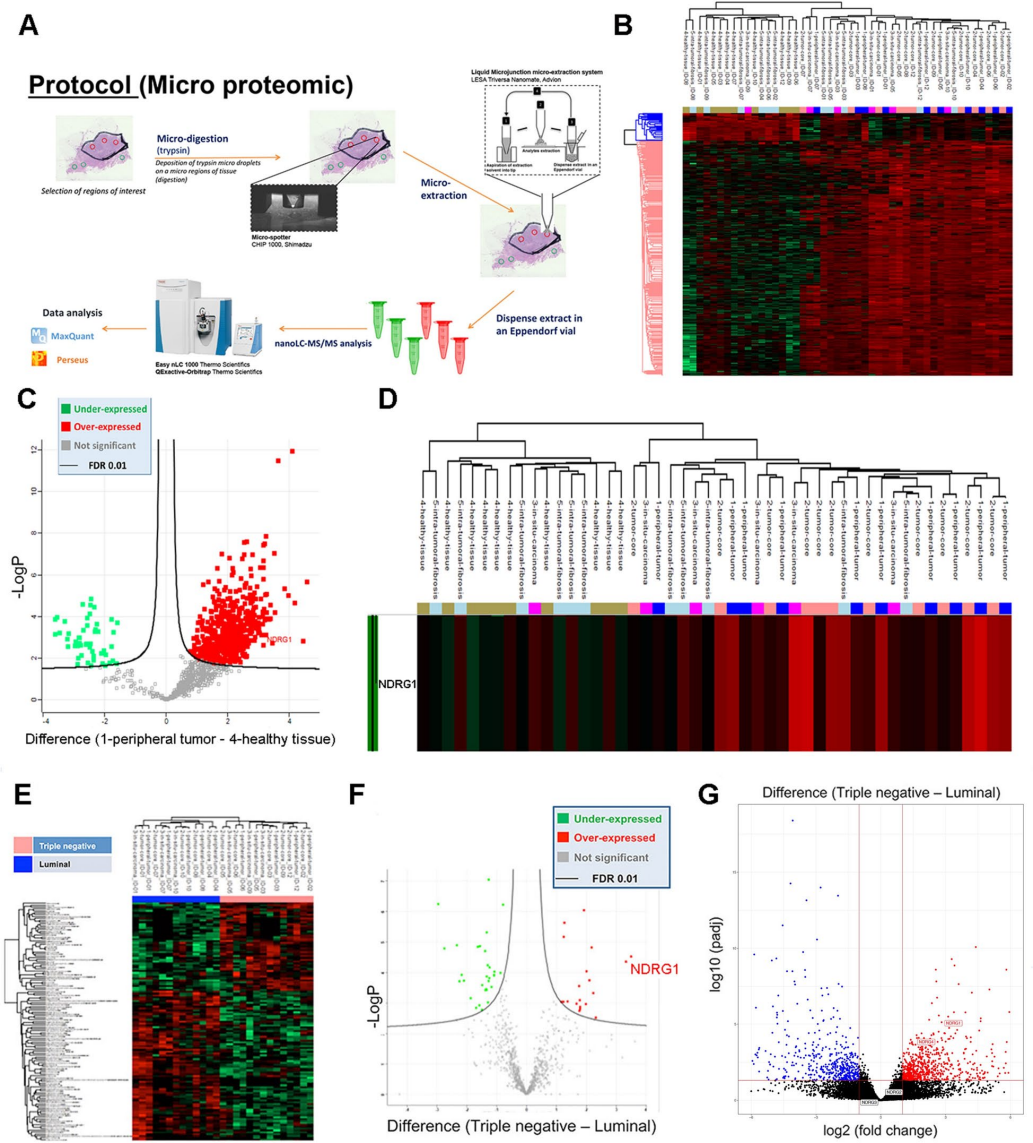
NDRG1 is highly expressed in breast cancer samples and TNBC subtypes. **A** Schematic representation of micro-proteomics workflow. Overview of trypsin deposition, protein extraction, and LC–MS/MS analysis. **B** The heat map based on Euclidean distance showed a significant separation between the healthy and tumor sections. The color scale ranges from red to green (highest to lowest relative expression). Each column of the heat map represents an independent sample and each row represents a specific protein. **C** Volcano plot of healthy vs tumor sections, from Perseus software. Significant proteins are determined using permutation-based FDR calculation with high confidence of 0.01 (solid line). Thresholds are displayed in the graph. **D** Spatial expression of NDRG1 in healthy and tumor sections. **E** The heat map based on Euclidean distance showed a significant separation between the Luminal and TNBC samples. The color scale ranges from red to green (highest to lowest relative expression). Each column of the heat map represents an independent sample and each row represents a specific protein. **F** Volcano plot of TNBCs vs Luminal, from Perseus software. The luminal group is chosen as a negative control. Significant proteins are determined using permutation-based FDR calculation with high confidence of 0.01 (solid line). Thresholds are displayed in the graph. **G** Differential expression of NDRG1 between Luminal A and TNBC samples analyzed by RNA seq

Twelve FFPE tumor sections consisting of 6 estrogen receptor (ER) and/or progesterone receptor (PgR) positive cases, and 6 triple negative breast cancer (TNBC) cases, were spatially analyzed by microproteomics, to provide an unsupervised and unlabeled in-depth proteomic profiling of BC spatial heterogeneity. In detail, a microproteomic mass spectrometry (MS)-based label-free quantification strategy was adopted. This approach combines trypsin micro-digestion, micro-extraction of peptides, and LC–MS/MS analysis (Q Exactive) followed by data analysis in MaxQuant and Perseus (Fig. 1A). Five different tissue sections were analyzed as judged by histology: peripheral tumor, tumor core, in situ carcinoma, healthy tissue, and intra-tumoral fibrosis. Hierarchical clustering analysis revealed a significant difference between tumor and healthy sections. As shown in Fig. 1B, two clusters of differentially expressed proteins were identified: cluster 1 containing 48 proteins, and cluster 2 containing 409 proteins. Specifically, Gene Ontology (GO) enrichment analysis indicated an overrepresentation of KEGG/Reactome pathways and biological processes related to the cellular response to stress including regulation of cellular response to stress, response to endoplasmic reticulum stress, response to oxidative stress, metabolism and protein processing in the endoplasmic reticulum (Table S2 and Fig. S1A). All of these pathways characterize tumor tissues that are subjected to a robust extrinsic and intrinsic stress response with a potential role in cancer growth, progression, and response to therapy [19]. Out of the differentially expressed proteins, NDRG1 emerged as one of the most significantly modulated between healthy and tumor samples. NDRG1 belongs to the group of proteins that respond to stress [20] (Supplementary MS/MS Data 1, Fig. 1), and was found to be strikingly upregulated in tumor samples compared to normal counterparts (Fig. 1C, D). Moreover, spatial proteomics analysis revealed that NDRG1 was most abundant in the tumor core, peripheral tumor, and in situ carcinoma compared to intra-tumoral fibrosis and healthy tissue (Fig. 1D).

Next, we examined proteomic data to reveal biomarkers or biomarker panels able to successfully segregate the tumour samples according to their molecular subtypes. We performed Perseus analysis of TNBC (n = 6) and Luminal (n = 6) tumors. The analysis revealed two subtypes with a distinct signature (Fig. 1E). As shown in Fig. 1E, a total of 127 proteins showed significantly altered levels of expression in the two subtypes by liquid chromatography–mass spectrometry/mass spectrometry (LC–MS/MS). Out of these proteins, 80 resulted to be upregulated in Luminal samples and 47 were upregulated in TNBC. Specifically, TNBC tumors were characterized by increased expression of epithelial mesenchymal transition (EMT) markers correlated with tumorigenesis and metastasis (including ACTN4, ANXA1, LDHB, and VIM), and enrichment of biological processes related to cell differentiation (false discovery rate, FDR = 1.14e−05) and response to stress (FDR = 0.0088), two key features of TNBC tumors. Both biological processes involve NDRG1, that was found to be expressed at significantly higher levels in TNBCs compared to Luminal samples (p = 4.35E−05, fold change, FC = 10) (Fig. 1F). The observed results were also confirmed at the mRNA level. RNAseq analysis of the same tumor cohort confirmed the overexpression of *NDRG1* in TNBC compared to Luminal samples according both to the PAM50 classification (FDR = 1.25E−08, log_2_FC = 3.36) and the immunohistochemical classification (FDR = 1.997E−05, log_2_FC = 3.16) (Fig. 1G). NDRG1 belongs to a family of four members, whose expression and functional role in BC progression remains unclear. We therefore investigated the expression of NDRG2, NDRG3 and NDRG4 in our MS/MS and RNAseq datasets, and found that their expression levels were particularly low, hampering their detection in our experimental workflow by MS/MS proteomics. When exploring the RNAseq data, only *NDRG4* resulted significant altered between cancer and normal conditions (PAM50: FDR = 1.47E−04 log_2_FC = 1.893, IHC: FDR = 3.44E−04 log_2_FC = 1.894), while *NDRG2* and *NDRG3* were not found to be differentially expressed between the two subgroups. Overall, these data reveal that TNBCs are characterized by elevated stress pathways and that NDRG1 can be one of the most reliable marker of these stress conditions.

**Fig. 2:**
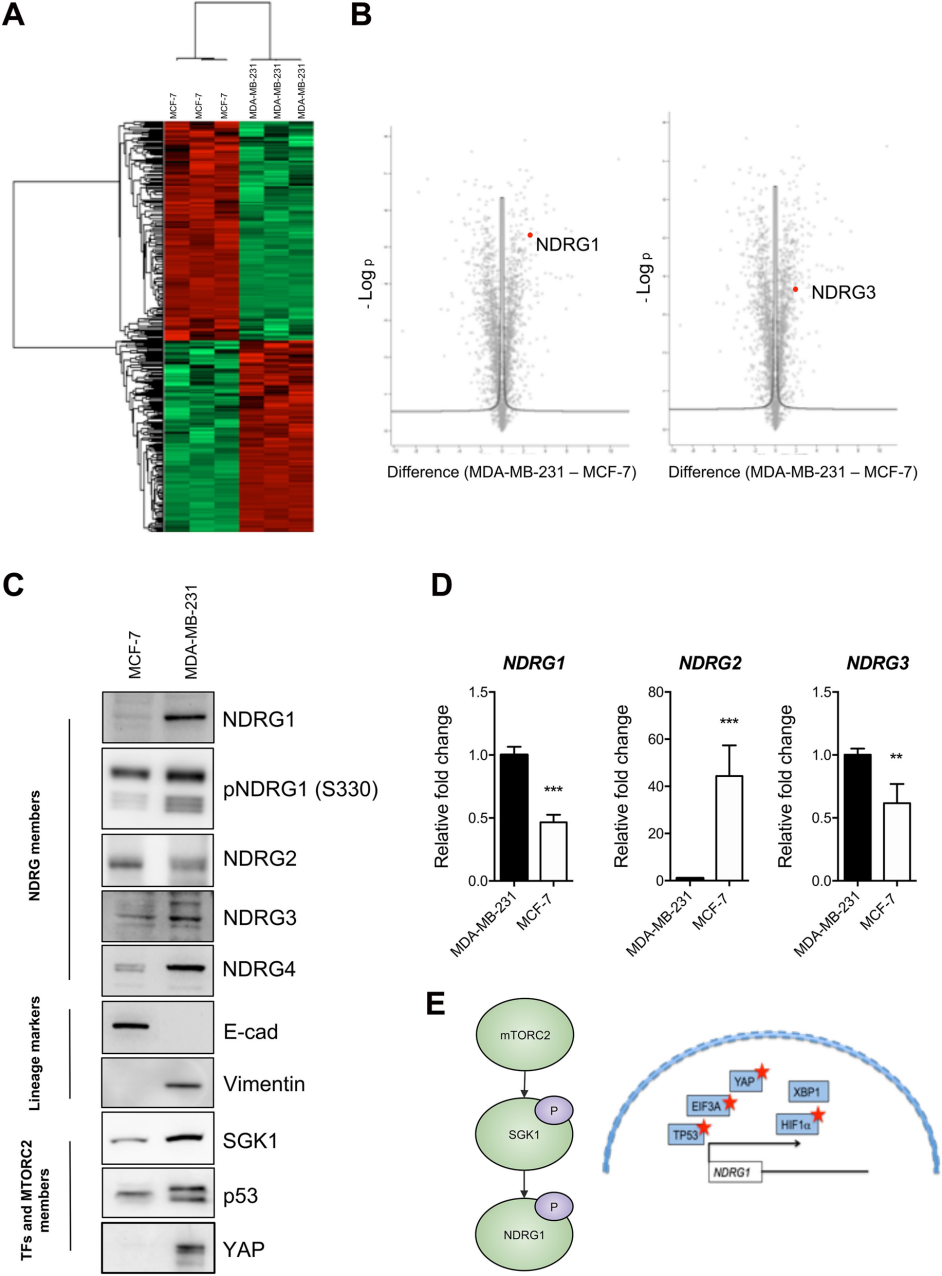
NDRG1 is highly expressed in a TNBC model in vitro. **A** The heat map based on Euclidean distance showed a significant separation between the Luminal model MCF-7 and the TNBC model MDA-MB-231. The color scale ranges from red to green (highest to lowest relative expression). Each column of the heat map represents an independent sample and each row represents a specific protein. **B** Volcano plot of MDA-MB-231 vs MCF-7, from Perseus software. The differential expression of NDRG1 and NDRG3 is highlighted. **C** Western blotting analysis for NDRG1, phospho-NDRG1 (S330), NDRG2, NDRG3, NDRG4, E-cadherin, Vimentin, SGK1, p53 and YAP of lysates obtained from MDA-MB-231 and MCF-7 cells. D) RT-qPCR of *NDRG1*, *NDRG2*, and *NDRG3* mRNAs in MDA-MB-231 and MCF-7 cells. The p-value was calculated using the Student’s t-test. The error bar represents ± SD. p-value ** < 0.01, *** < 0.001. **E** Posttranslational and transcriptional mechanisms of NDRG1 regulation. Left. The mTORC2/SGK1 pathway regulates the phosphorylation of NDRG1. Right. The red stars indicate differentially expressed proteins involved in the regulation of *NDRG1* expression and identified by MS/MS. The lists of proteins detected are presented in Supplementary MS/MS Data 2

In agreement with data obtained using tissue samples, LC–MS/MS analysis of MCF-7 (Luminal) and MDA-MB-231 (TNBC) cells revealed a significant enrichment of biological processes and KEGG pathways associated with stress in the TNBC model [cellular response to stress (p = 2.8E−03), response to endoplasmic reticulum stress (p = 6.5E−02), cellular response to oxidative stress (p = 7.4E−02), and protein processing in the endoplasmic reticulum (p = 2.7E−04) (Fig. S1B)]. In line with this, among the most significantly modulated proteins, NDRG1 was found in the cluster of MDA-MB-231 upregulated proteins (Fig. 2A, B). In addition, we identified NDRG3 as significantly upregulated in the TNBC model. Again, the low abundance of NDRG2 and NDRG4 members limits their detection by MS/MS. These findings as well as the expression of other NDRG members were confirmed and investigated by western blotting and qPCR (Fig. 2C, D). Data demonstrated a different expression of the NDRG2 and NDRG4 isoforms, together with an increase of the phosphorylated form of NDRG1, between the two cell lines. Consistent with the increased phosphorylation, mRNA, and protein levels of NDRG1 in the MDA-MB-231 model compared to MCF-7, we observed increased levels of the kinase SGK1, and the transcription factors p53 and YAP in the TNBC model (Fig. 2C). Moreover, LC–MS/MS data confirmed the upregulation of p53 and YAP observed via western blot and identifies other possible regulators of NDRG1 as more highly expressed in the TNBC model including EIF3A and the HIF pathway (Supplementary MS/MS Data 2 and Fig. 2E). In fact, GO analysis by DAVID identified HIF pathway among those pathways significantly enriched in MDA-MB-231 cells (Fig. S1C). As TNBC tumors express molecular markers of EMT, to understand whether the differential expression of NDRG1 is linked to the different EMT state of MDA-MB-231 and MCF-7 cells, we included a Luminal B model in the proteomic analysis and used the GEO dataset to query the database on the possible correlation between NDRG1 and EMT markers. Overall, the Luminal B cell line MDA-MB-361 shows expression levels of NDRG1 and NDRG3 comparable to those of the TNBC model (Fig. S2A, B). Furthermore, except for an increase of NDRG1/2/3/4 expression observed in the MCF-7 model after Slug over-expression, the analysis of the GEO24202 and GSE41313 datasets does not correlate the expression of NDRG1 and the other members of the NDRG family with the process of EMT in BC (Fig. S2C–E).

### NDRG1 is an independent prognostic marker of disease outcome

**Fig. 3:**
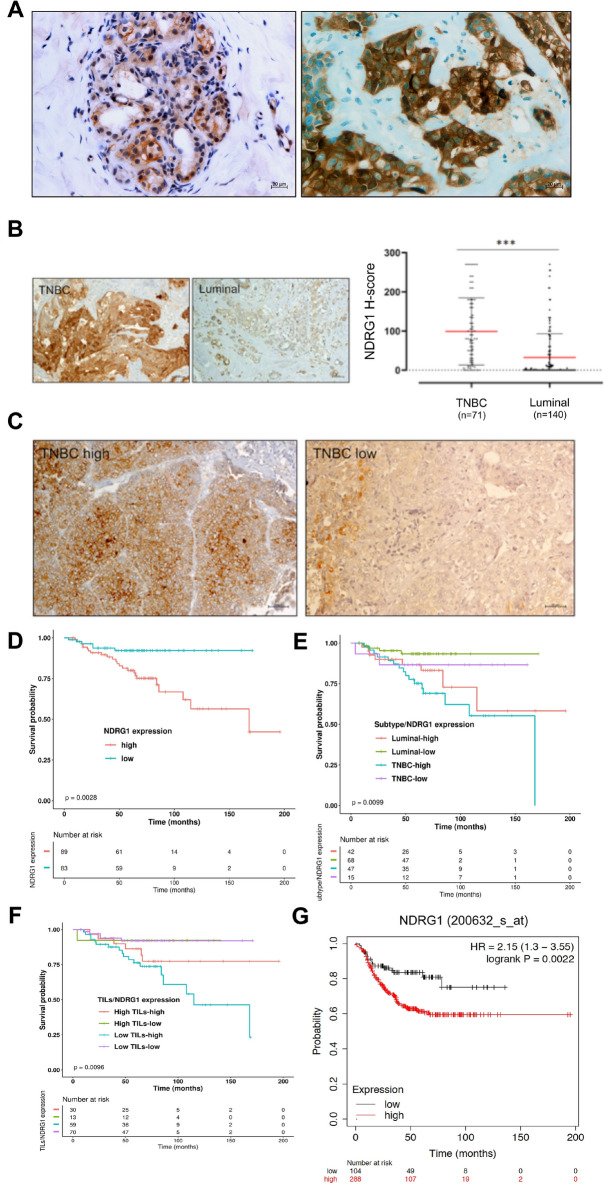
NDRG1 is over-expressed in BC samples. **A** Differential NDRG1 expression in BC. Compared to normal breast tissue (left), invasive ductal carcinoma (right) shows a marked increase in NDRG1 expression (original magnification, × 200). Scale bar = 20 µm. Images were obtained on an Axion Image 2 upright microscope (Zeiss, Oberkochen, Germany) with an Axiocam 512 color camera. **B** On the left, representative images of immunohistochemical staining in BC tissues. The iamge displays the representative expression of NDRG1 in TNBC and luminal phenotype. (original magnification, × 200). Scale bar = 50 µm. Images were obtained on an Axion Image 2 upright microscope (Zeiss, Oberkochen, Germany) with an Axiocam 512 color camera. On the right, expression levels of NDRG1 in TNBCs compared with Luminal phenotype. Values are expressed as the median (horizontal red line in each box). Dots indicate outliers. ***p < 0.001. **C** Representative images of immunohistochemical staining in the TNBC subgroups. On the left, a case with high NDRG1 expression; on the right, a case with low NDRG1 expression. Scale bar = 50 µm. Kaplan–Maier curve analysis and log-rank test. **D** Kaplan–Maier curve for disease-free survival (DFS) according to high NDRG1 versus low expression in BC patients (p = 0.0028). **E** DFS in the subgroups of Luminal (Luminal A and B) and TNBC tumors, according to high and low NDRG1 expression (p = 0.0099). **F** DFS of TILs/NDRG1 co-expression showed that the patients with low TILs/high NDRG1 tumors had a worse DFS with respect to the other phenotypes considered (p = 0.0096). **G** The Kaplan‒Meier Plotter website was used to investigate the relationship between DRG1 expression and survival probability in TNBC human samples (n(high) = 288, n(low) = 104)

We further retrospectively analyzed NDRG1 protein expression levels in a total of n = 211 breast tumor samples (n = 71 TNBCs and n = 140 Luminal BCs) and normal regions non adjacent to the tumor (n = 10, distance > 2 cm) by IHC. NDRG1 was detected in 134/211 patients (63.5%); it was mainly expressed in the cytoplasm (44%), in the cell membrane (29%), in the cell membrane and cytoplasm simultaneously (27%), and in the nucleus with membrane and cytoplasm (9%) (Fig. S3A, B, C). Nuclear localization of NDRG1 was largely observed in the samples with the highest staining intensity (3 +), with a range of expression from 1 to 20% of positive cells. To confirm NDRG1 localization, a dual fluorescence immunostaining was performed on tissue sections (Fig. S3D). In normal breast tissue regions, NDRG1 showed low levels of expression and mainly localized in the cell membrane. On the other hand, its expression was significantly increased in tumor regions (Fig. 3A). Further analysis based on molecular subtypes demonstrated that NDRG1 was expressed at higher levels in TNBC than in Luminal BC samples (p ≤ 0.0001), confirming LC–MS/MS and RNAseq results (Fig. 3B). Table S3 shows the relationship between NDRG1 and the clinicopathological characteristics. NDRG1 over-expression was observed in invasive ductal carcinoma (IDC; p = 0.01). Further, NDRG1 expression was positively correlated to the histological Grade (G3) and with Ki67 (p < 0.0001), and inversely correlated to the expression of ER (p < 0.0001), PgR (p < 0.0001), androgen receptor (AR) (p = 0.0182), Her2 status (p = 0.009) and tumour-infiltrating lymphocytes (TILs) presence (p = 0.008). NDRG1 was not associated with age at diagnosis (p = 0.24). Moreover, two subtypes of BC samples could be identified according to the expression of NDRG1: NDRG1-high (H-score median value > 10) and NDRG1-low (H-score median value ≤ 10) (Fig. 3C). According to univariate analysis, high NDRG1 in tumors cells was associated with a worse disease-free survival (DFS) compared to tumors with low NDRG1 expression (hazard ratio, HR = 2.59; 95% confidence interval, CI: 1.20, 5.59; p = 0.016). We also found a significant association between Nodal positivity and TNBCs phenotype with poorer DFS (p = 0.023, p = 0.039, respectively) (Table S4). Kaplan–Meier curves confirmed that BC patients with high expression of NDRG1 had a worse DFS than patients with low expression of the protein (p = 0.0028, Fig. 3D). Taking into account the subgroups of Luminal tumors (Luminal A and B) and TNBCs, we found that TNBC patients with high NDRG1 expression had the worst DFS (p = 0.0099, Fig. 3E). Furthermore, considering TILs/NDRG1 co-expression in the BC group we observed that the patients with low TILs/high NDRG1 tumors had a worse DFS with respect to the other phenotypes considered (p = 0.0096; Fig. 3F) and we observed the same trend in the TNBCs group, although without statistical significance (data not shown). In multivariate analysis, high NDRG1 expression and positive node status were independently associated with poorer DFS. In detail, patients with low NRDG1 showed HR = 0.45 (95% CI 0.2 ÷ 1.02, p = 0.05) and those with nodal positivity had HR = 2.28 (95% CI 1.13 ÷ 4.58, p = 0.02) (Table S5). Our results were mainly focused on DFS, and not overall survival (OS), due to the low number of deaths in our cohort. DFS of TNBC patients was also analyzed on an independent case series of n = 392 patients using the Kaplan Meier plotter tool (www.kmplot.com). As shown in Fig. 3G, high *NDRG1* mRNA expression was found to be highly correlated to poor DFS. We then investigated the relationship between NDRG1 and BC patients' survival in relation to its different localization (membrane, cytoplasm, and nucleus). The high NDRG1 expression in the cytoplasm and nuclear compartment was related to worse survival, although not statistically significant, both in BC and in the TNBC sub-groups (data not shown). This appears in line with previous reports showing a re-localization of NDRG1 from the plasma membrane to the cytoplasm and nucleus in response to induction of hypoxia, a condition frequently occurring within the tumor microenvironment, that ultimately allows tumors to survive and become resistant to various therapeutic regimens [21]. In the whole BC cohort, patients with tumors positive for membranous expression of NDRG1 had higher DFS than patients with tumors negative for membranous expression of NDRG1 (p = 0.04). Interestingly, patients with tumors positive for cytoplasmic NDRG1 expression showed a lower DFS than patients with tumors negative for cytoplasmic NDRG1 (p = 0.05). No difference was observed in DFS for the patients with positive versus negative nuclear NDRG1 expression (Fig. S3E). Similar results were observed in the sub-group of TNBCs, although not statistically significant (data not shown), probably due to the limited sample size.

To explore the prognostic/predictive potential of NDRG1, the TCGA-BRCA dataset was used. In detail, a cohort including 707 patients with luminal BC and 174 patients with basal BC was selected. NDRG1 expression data was retrieved from transcriptomic profiling through RNAseq and it was dichotomized according to median value. Kaplan–Meier curves were generated according to molecular subtype (Luminal/Basal) and NDRG1 high/low expression and then they were statistically compared. The results highlighted a significantly different DFS (p = 0.0078). In particular, pairwise comparison showed that patients with Basal-low BC had a poorer outcome than patients with Luminal-high BC (p = 0.027). Patients with Basal BC with low expression of NDRG1 had a shorter DFS than those with high expression (p = 0.032) (Fig. S4A). However, multivariate Cox-hazard regression analysis did not confirm the independent predictive role of NDRG1 (Fig. S4B). Kaplan–Meier OS curves were also significantly different (p = 0.03). Luminal BC patients with high NDRG1 expression had a poorer OS than BC patients with low NDRG1 expression (Fig. S4C) and, indeed, multivariate Cox-Hazard regression analysis confirmed that NDRG1 expression is a prognostic independent factor (HR: 0.58; 95% CI 0.35–0.99; p = 0.04) (Fig. S4D).

### PKC activation regulates NDRG1 expression

**Fig. 4:**
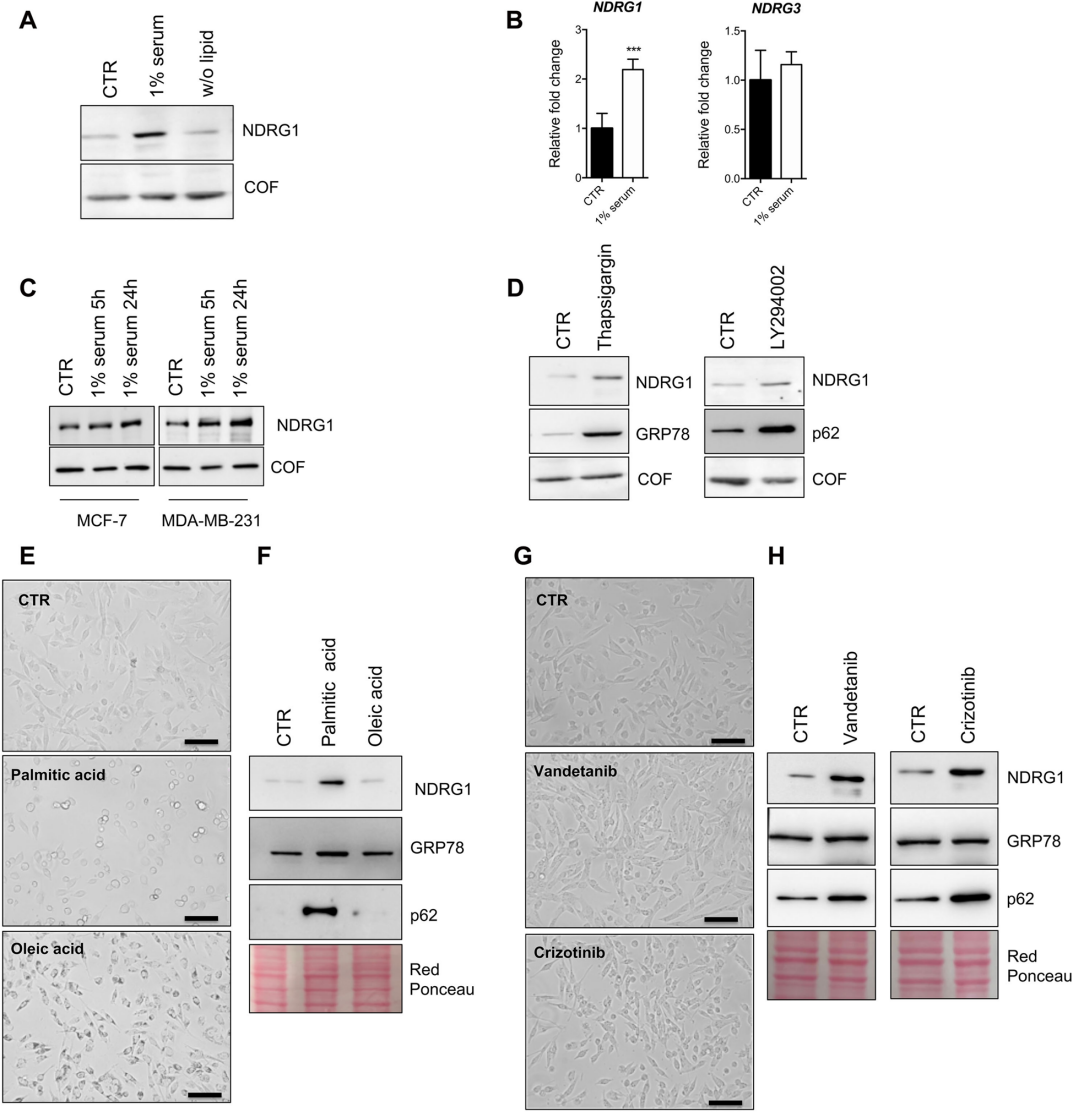
NDRG1 is a stress-responsive protein. **A** Western blotting analysis for NDRG1 of lysates obtained from MDA-MB-231 cells exposed to 1% serum or maintained in a medium without lipids for 24 h. Cofilin was used as a loading control. **B** RT-qPCR of *NDRG1* and *NDRG3* mRNAs in control and MDA-MB-231 cells exposed to 1% serum for 24 h. The p-value was calculated using the Student’s t-test. The error bar represents ± SD. p-value *** < 0.001. **C** Western blotting analysis for NDRG1 of lysates obtained from MCF-7 and MDA-MB-231 cells exposed to 1% serum for 5 h or 24 h. Cofilin was used as a loading control. **D** Western blotting analysis for NDRG1, GRP78 and p62 of lysates obtained from MDA-MB-231 cells exposed to thapsigargin (1 μM) or LY294002 (10 μM) for 24 h. Cofilin was used as a loading control. **E** Representative images of MDA-MB-231 cells cultured in a normal condition medium or treated palimitic acid (200 μM), or oleic acid (200 μM) for 24 h. Images were acquired using an inverted wide-field microscope (EVOS FLoid Cell Imaging Station, Thermo). Scale bar 100 μm. **F** Western blotting analysis for NDRG1, GRP78 and p62 of lysates obtained from MDA-MB-231 cells exposed to palmitic acid or oleic acid at the concentration of 200 μM for 24 h. **G** Representative images of MDA-MB-231 cells cultured in a normal condition medium or treated with Vandetanib (10 μM), or Crizotinib (10 μM) for 24 h. Images were acquired using an inverted wide-field microscope (EVOS FLoid Cell Imaging Station, Thermo). Scale bar 100 μm. **H** Western blotting analysis for NDRG1, GRP78 and p62 of lysates obtained from MDA-MB-231 cells exposed to Vandetanib and Crizotinib treatment at the concentration of 10 μM for 24 h

Ex-vivo proteome comparison between Luminal and TNBC tissues, revealed an overrepresentation of proteins associated with stress stimuli. These data suggest that NDRG1 likely has a functional association with these processes. In fact, we observed significant up-regulation of NDRG1 in MDA-MB-231 cells under stress conditions obtained by 24 h incubation under serum deprivation (1% of serum) but not under lipids deprivation (Fig. 4A). This response is specific for NDRG1, as other isoforms such as NDRG3 do not show any differential expression upon stress (Fig. 4B). The increased expression of NDRG1 is already observed after 5 h of treatment in both MDA-MB-231 and MCF-7 cells (Fig. 4C). The effects of extracellular stress conditions can converge at the cellular level on the activation of mechanisms of response including endoplasmic reticulum (ER) stress and autophagy. The activation of ER stress by thapsigargin and the modulation of autophagic flux by LY2949002 both up-regulate NDRG1 (Fig. 4D). Similarly, metabolic or chemical stresses that modulate ER and autophagy processes lead to overexpression of NDRG1 (Fig. 4E–H).

**Fig. 5:**
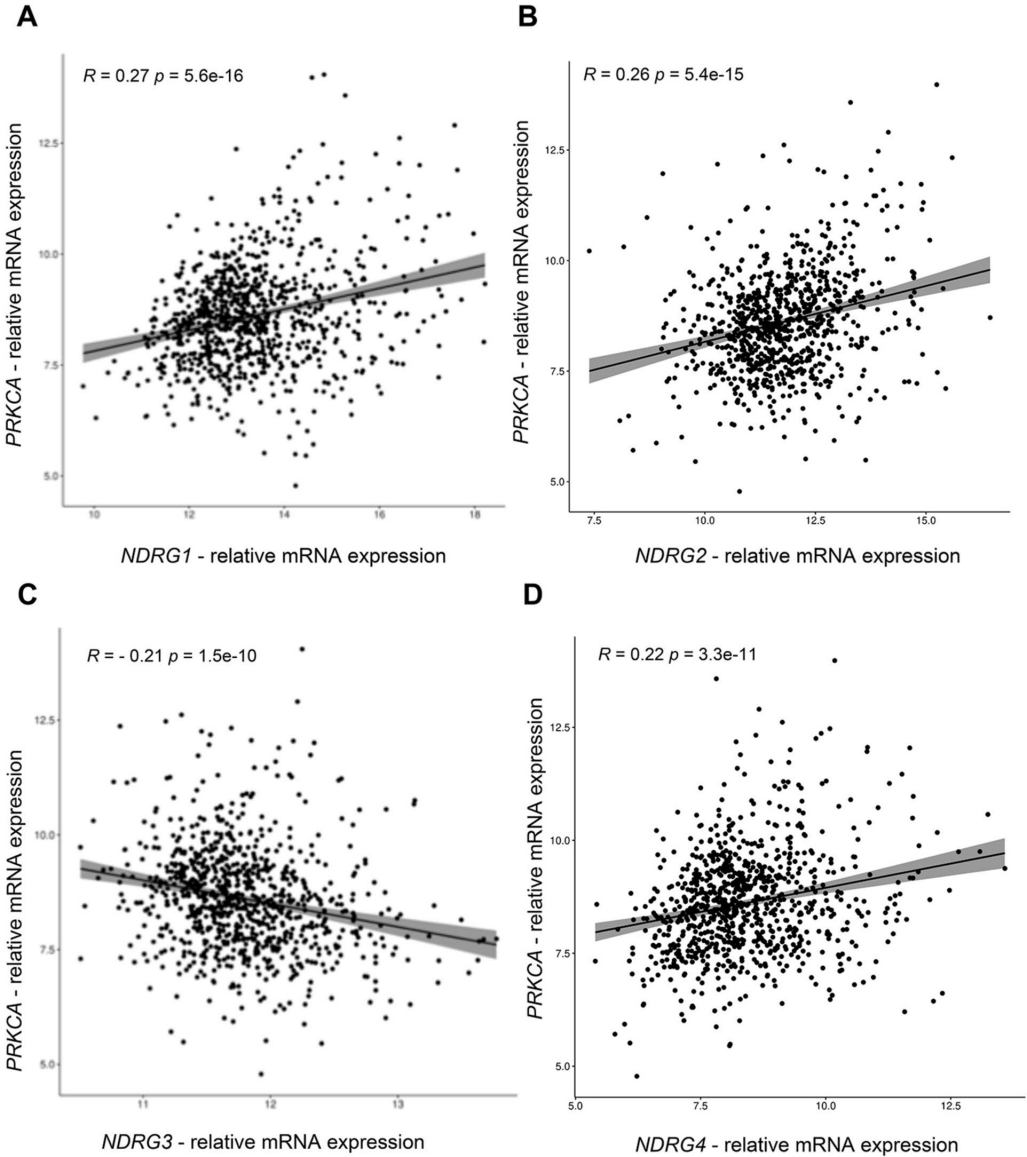
Results of TCGA data analysis. Correlation of *PRKCA*, *NDRG1*, *NDRG2, NDRG3* and *NDRG4* mRNAs expression in BC samples of the TCGA database

**Fig. 6:**
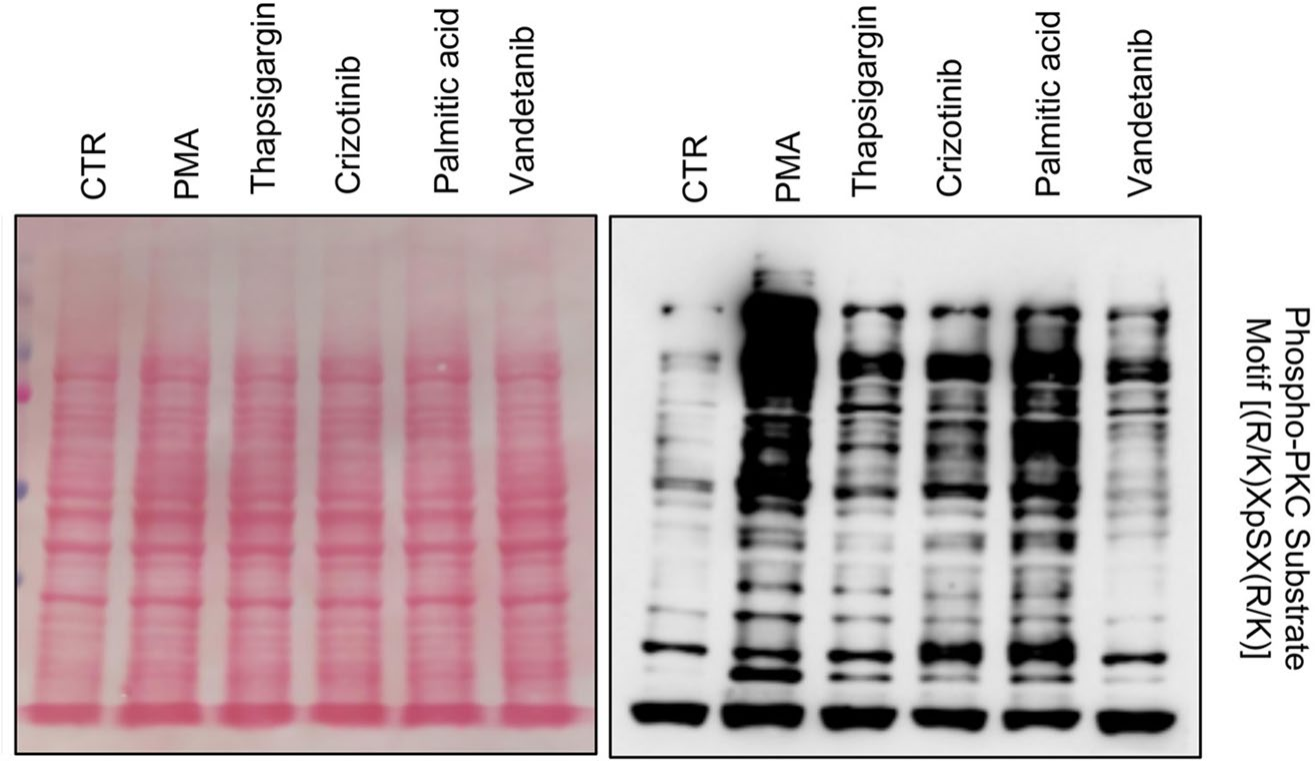
Different stress conditions activate PKC. Western blotting analysis for Phospho-PKC Substrate Motif [(R/K)XpSX(R/K)] of lysates obtained from MDA-MB-231 cells exposed to PMA, thapsigargin, Crizotinib, palmitic acid, and Vandetanib for 30 min

**Fig. 7:**
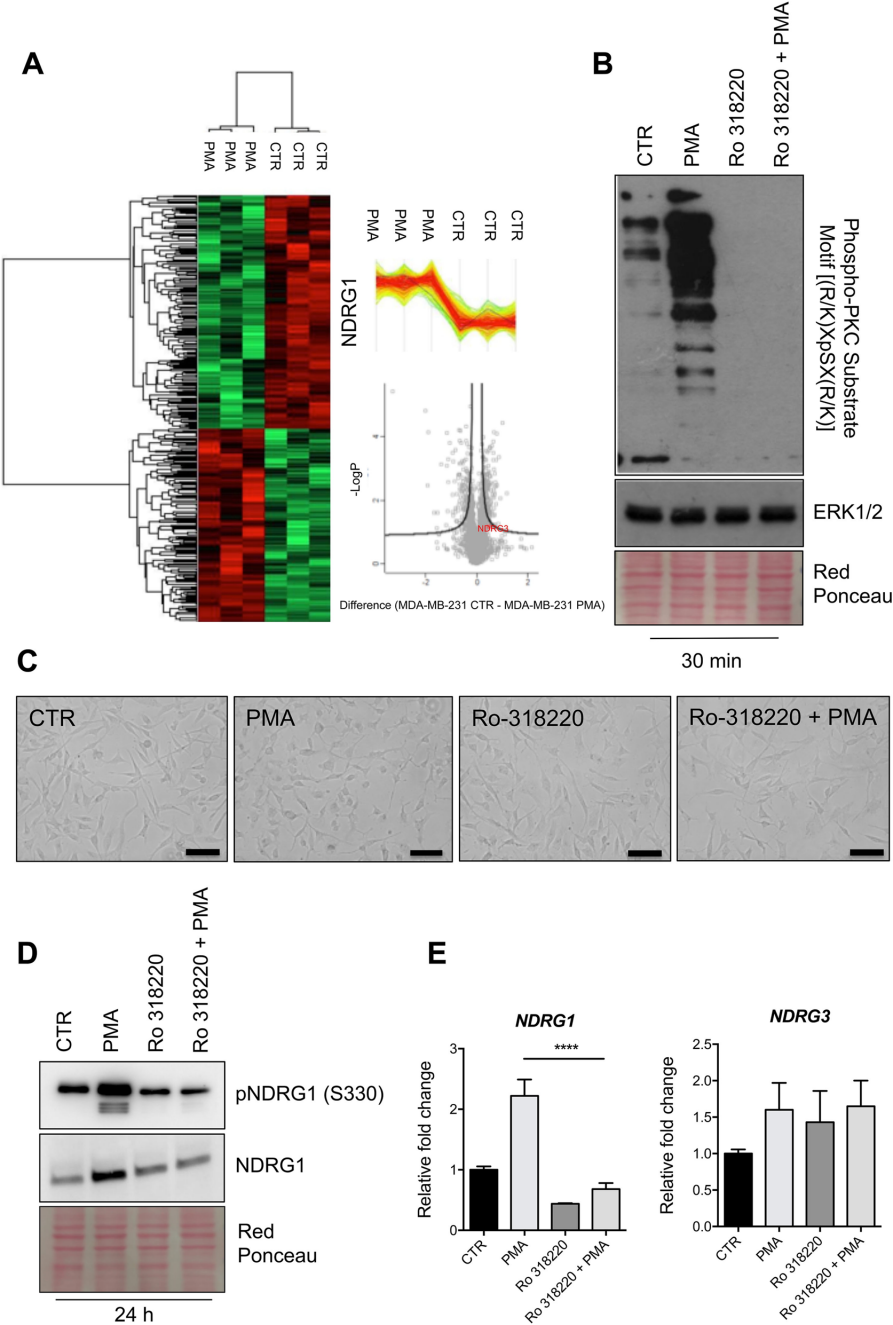
Mass spectrometry analysis of PMA-treated MDA-MB-231 cells. **A** The heat map based on Euclidean distance showed a significant separation between the control and PMA-treated MDA-MB-231 cells. The color scale ranges from red to green (highest to lowest relative expression). Each column of the heat map represents an independent sample and each row represents a specific protein. The window contains the expression profiles of NDRG1. **B** Western blotting analysis for Phospho-PKC Substrate Motif [(R/K)XpSX(R/K)] of lysates obtained from MDA-MB-231 cells exposed to PMA (100 nM), Ro318220 (1 μM) alone or in combination for 30 min. **C** Bright-field images of MDA-MD-231 cells treated with PMA (100 nM), Ro318220 (1 μM) alone or in combination for 24 h. Images were acquired using an inverted wide-field microscope (EVOS FLoid Cell Imaging Station, Thermo). Scale bar 100 μM. **D** Western blotting analysis for NDRG1 and phospho-NDRG1 of lysates obtained from MDA-MD-231 cells treated with PMA (100 nM), Ro318220 (1 μM) alone or in combination for 24 h. **E** RT-qPCR in MDA-MB-231 cells subjected to exposed to PMA (100 nM), Ro318220 (1 μM) alone or in combination for 24 h. The p-value was calculated using the ANOVA test. The error bar represents ± SD. p-value **** < 0.0001

It has been reported that protein kinase C (PKC) can be activated in a large variety of stress cues to mediate cell survival [22, 23], suggesting that the up-regulation of NDRG1 upon stress could be mediated through the PKC signaling network. First, we investigated whether a functional association between PKC and NDRG1 occurs in BC. Hence, we performed correlation analyses using the TCGA cohort. A significant positive correlation of *PRKCA* expression with *NDRG1* (R = 0.27, p = 5.6e−16), *NDRG2* (R = 0.26, p = 5.4e−15), and *NDRG4* (R = 0.22, p = 3.3e−11) was indeed revealed. On the other hand, a negative correlation was observed between *PRKCA* and *NDRG3* (R = − 0.21, p = 1.5e−10) (Fig. 5A–D). Second, we assessed the activation of PKC in MDA-MB-231 cells by thapsigargin, Crizotinib, palmitic acid, and Vandetanib. As shown in Fig. 6, the activation of PKC was confirmed by western blot using a phospho-PKC substrate antibody. Third, to experimentally assess that the activation of PKC leads to increased NDRG1 expression, we treated MDA-MB-231 cells with the PKC activator phorbol-12-myristate-13-acetate (PMA) and performed proteomic analysis of the corresponding protein lysates (Supplementary MS/MS Data 3, Fig. 7A). NDRG1, but not the expression of other NDRG isoforms, are significantly up-regulated after treatment. Next, we treated MDA-MB-231 cells with the PKC inhibitor Ro-318220 alone or in combination with PMA. PMA-induced activation of PKC (Fig. 7B), PMA-induced morphological alterations of the MDA-MB-231 cells (Fig. 7C), and NDRG1 and phospho-NDRG1 over-expression (Fig. 7D) were suppressed by this treatment. Moreover, the mRNA of *NDRG1* but not *NDRG3* was significantly up-regulated after PMA treatment (Fig. 7E). Altogether, these data strongly indicate a functional correlation between PKC and NDRG1 in TNBC cells.

Up-regulation of NDRG1 by PMA was also confirmed in additional BC cells. We observed a specific up-regulation of NDRG1, at the protein and mRNA level in MCF-7 and Hs 578 T cells treated with PMA for 24 h (Fig. S5A, B). As a negative control, T47D cells displayed a complete absence of the NDRG1 after PMA treatment (Fig. S5A). In our previous work, we analysed the proteome of MCF-7 cells and compared it to the proteome of MCF-7 treated with PMA. Consistent with results shown in Fig. S5A, the analysis of our proteomic dataset confirmed that NDRG1 was among the proteins up-regulated after PMA treatment (Fig. S5C) Moreover, we observed that treatment of the Huh7 hepatocellular carcinoma cell line, and of the HCT-15 and HCT-116 colon cancer cell lines with PMA led to an increased expression of NDRG1 (Fig. S5D). These data extend the first evidence obtained in TNBC cells, and show that the PKC → NDRG1 axis is widespread across various tumor types.

**Fig. 8:**
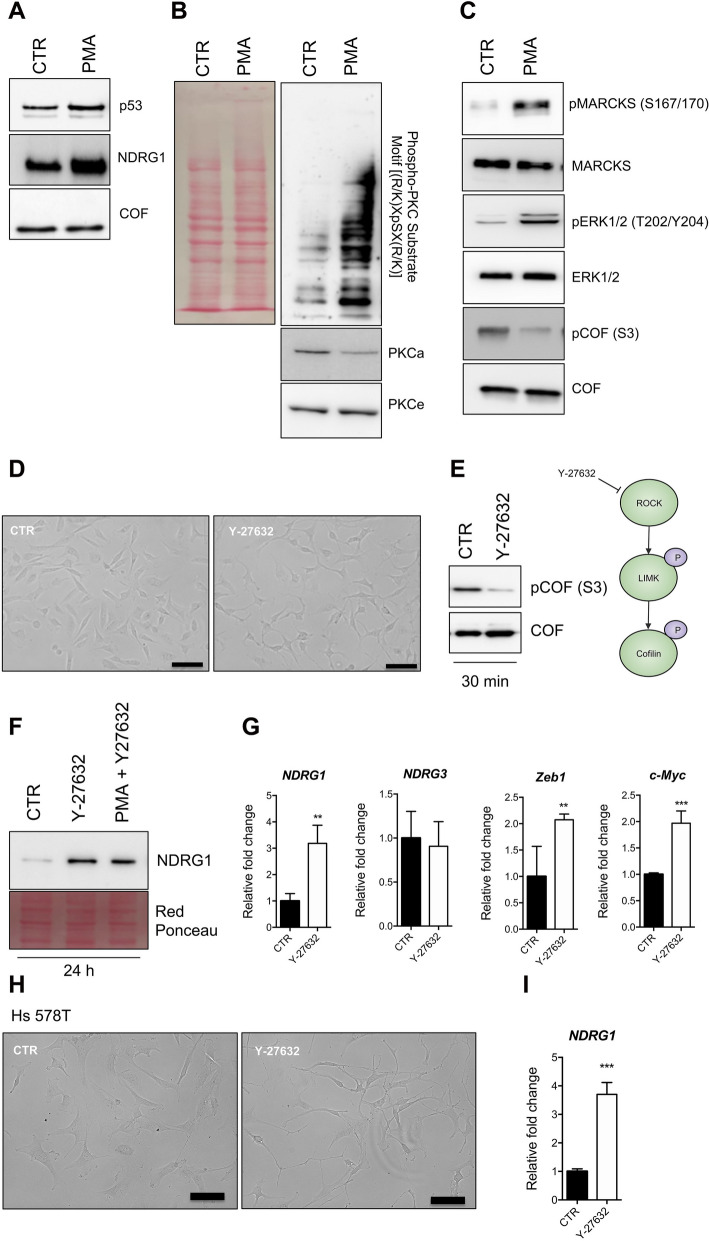
Mechanisms for NDRG1 regulation by PKC. **A** Western blotting analysis for p53, NDRG1 of lysates obtained from MDA-MB-231 cells exposed to PMA (100 nM) for 24 h. Cofilin was used as a loading control. **B** and **C** Western blotting analysis for phospho-PKC Substrate Motif [(R/K)XpSX(R/K)], PKCα, PKCε, phospho-MARCKS (Ser167/170), MARCKS, phospho-Erk1/2, Erk1/2, phospho-Cofilin, and Cofilin of lysates obtained from MDA-MB-231 cells exposed to PMA (100 nM) for 30 min. Cofilin was used as a loading control. ROCK inhibition induces NDRG1 expression. **D** Representative images of MDA-MB-231 cells cultured in a normal condition medium or treated with 100 nM Y-27632 for 24 h. Images were acquired using an inverted wide-field microscope (EVOS FLoid Cell Imaging Station, Thermo). Scale bar 100 μm. E) Western blotting analysis for phospho-Cofilin, and Cofilin of lysates obtained from MDA-MB-231 cells exposed to Y-27632 (10 μM), for 30 min. **F** Western blotting analysis for NDRG1 of lysates obtained from MDA-MB-231 cells exposed to PMA (100 nM), Y-27632 (10 μM) alone or in combination for 24 h. **G** RT-qPCR of *NDRG1*, *NDRG3*, *Zeb1* and *c-Myc* mRNAs in control and MDA-MB-231 cells exposed to Y-27632 (10 μM) for 24 h. The p-value was calculated using the Student’s t-test. The error bar represents ± SD. p-value ** < 0.01, *** < 0.001. **H** Representative images of Hs 578 T cells cultured in a normal condition medium or treated with 100 nM Y-27632 for 24 h. Images were acquired using an inverted wide-field microscope (EVOS FLoid Cell Imaging Station, Thermo). Scale bar 100 μm. **I** RT-qPCR of *NDRG1* mRNA in control and Hs 578 T cells exposed to Y-27632 (10 μM) for 24 h. The p-value was calculated using the Student’s t-test. The error bar represents ± SD. p-value *** < 0.001

To assess the molecular mechanisms of the PKC → NDRG1 axis, we initially focused on the most relevant transcription factors known to regulate NDRG1 expression, p53. We treated MDA-MB-231 cells with PMA for 24 h and analysed the expression of p53. PMA induced up-regulation of p53 (Fig. 8A). Moreover, p53 overexpression was observed after treatment with thapsigargin with a concomitant increase of NDRG1 at the protein and mRNA level (Fig. S6A, B). The up-regulation of p53 was measured upon treatment of MCF-7 cells with PMA (Fig. S6C), extending the observation done on the TNBC cell line MDA-MB-231.

To elucidate specific signaling mechanisms underlying the over-expression of NDRG1 after PKC activation, we treated MDA-MB-231 cells with PMA. We investigated the activation of PKC and the phosphorylation status of downstream kinases known to belong to pathways regulated by PKC (Fig. 8B, C). After 30 min of stimulation, PMA activated PKC, MARCKS and ERK1/2. In contrast, phospho-cofilin (pCOF) levels were reduced after PMA treatment (Fig. 8C).

Cofilin is a known substrate of the kinase ROCK and plays a key role in the modulation of actin cytoskeleton. Thus, as treatment with PMA leads to dephosphorylation of Cofilin, we assessed whether inhibiting ROCK is sufficient to induce NDRG1 up-regulation. To do this, we treated MDA-MB-231 cells with the ROCK inhibitor Y-27632 for 24 h. As shown in Fig. 8D, E, inhibition of ROCK induces morphological changes and reduces the phosphorylation levels of Cofilin in MDA-MB-231 cells. Interestingly, *NDRG1* levels, not *NDRG3*, were found to significantly increase after Y-27632 treatment (Fig. 8F, G). The effect of Y-27632 and PMA, however, was not synergic, as NDRG1 levels were comparable in cells treated with PMA and cells treated with PMA + Y-27632 (Fig. 8F). This suggests that both compounds converge on the same signaling node. Moreover, ROCK inhibition significantly increases mRNA levels of Zeb1 and c-Myc, two genes associated with the metastatic potential of BC. Similarly, Y-27632 treatment led to increase of the *NDRG1* mRNA expression in a second TNBC cell model (Hs 578 T, Fig. 8H).

### NDRG1 is required for breast cancer cell invasion in vitro

**Fig. 9:**
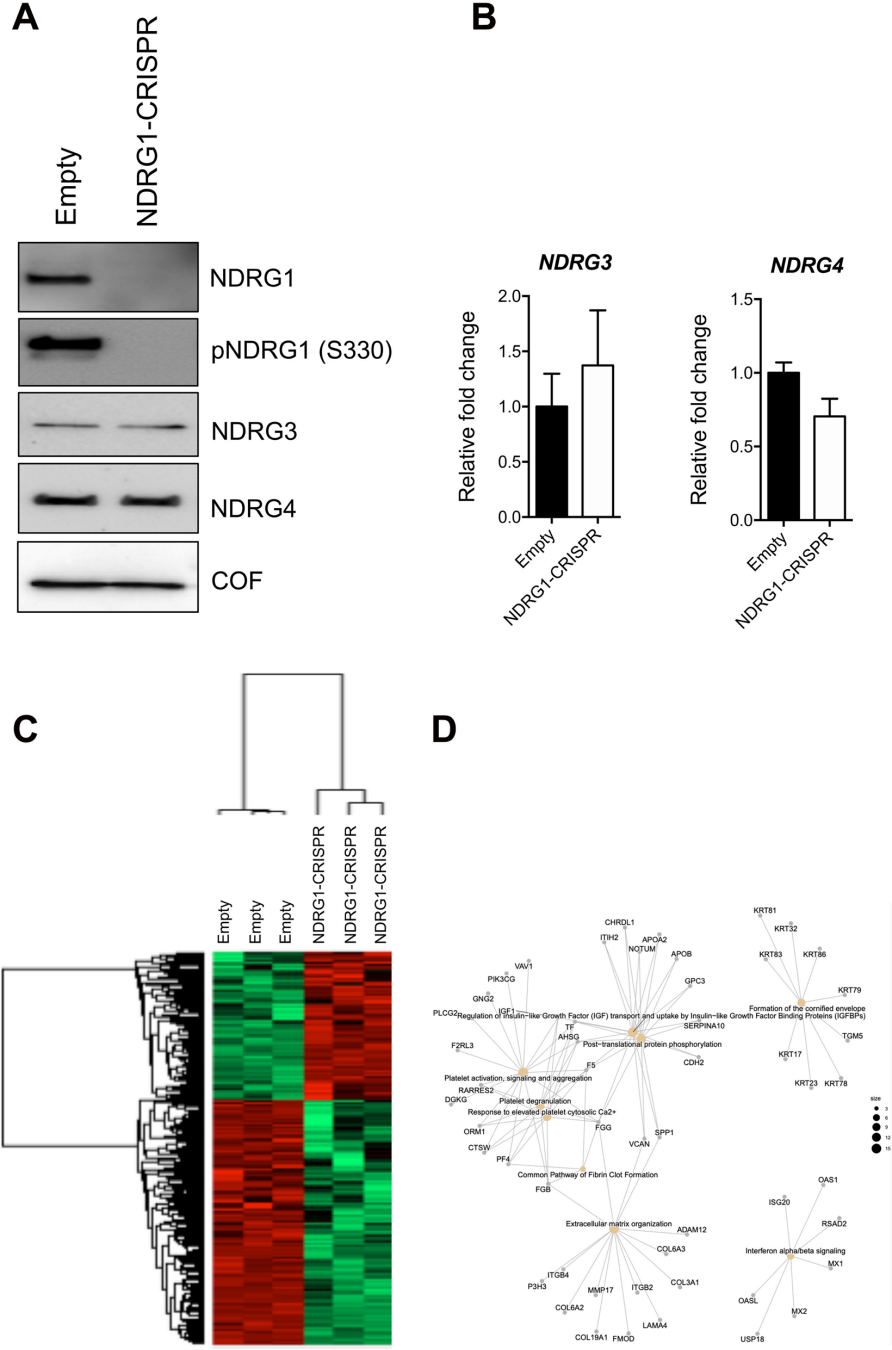
CRISPR/Cas9 knockdown of NDRG1. **A** Western blotting analysis for NDRG1, pNDRG1 (S330), NDRG3 and NDRG4 of lysates obtained from MDA-MB-231 Empty and NDRG1-CRISPR cells. Cofilin was used as a loading control. **B** RT-qPCR of *NDRG3* and *NDRG4* mRNAs in MDA-MB-231 Empty and NDRG1-CRISPR cells. CRISPR/Cas9 knockdown of NDRG1 modulates the proteome of MDA-MB-231 cells. **C** Heat map based on Euclidean distance showing a significant separation between the MDA-MB-231 Empty and NDRG1-CRISPR cells. Each row of the heat map represents a protein, and each column represents an independent sample. Two main clusters were identified from the hierarchical clustering, and their pattern is reported. **D** Enrichment maps of deregulated genes in Empty and NDRG1-CRISPR cells

To investigate the functional role of NDRG1 in the regulation of the invasive phenotype of TNBC cells, we decided to use a CRISPR/Cas9-based gene editing approach to inhibit the expression of *NDRG1* in MDA-MB-231 cells (Fig. 9A). We ruled out the chance that a compensatory mechanism could occur upon inhibition of NDRG1 expression, as the expression of other NDRG members, such as NDRG3, and NDRG4, was verified to be unchanged (Fig. 9A, B).

**Fig. 10:**
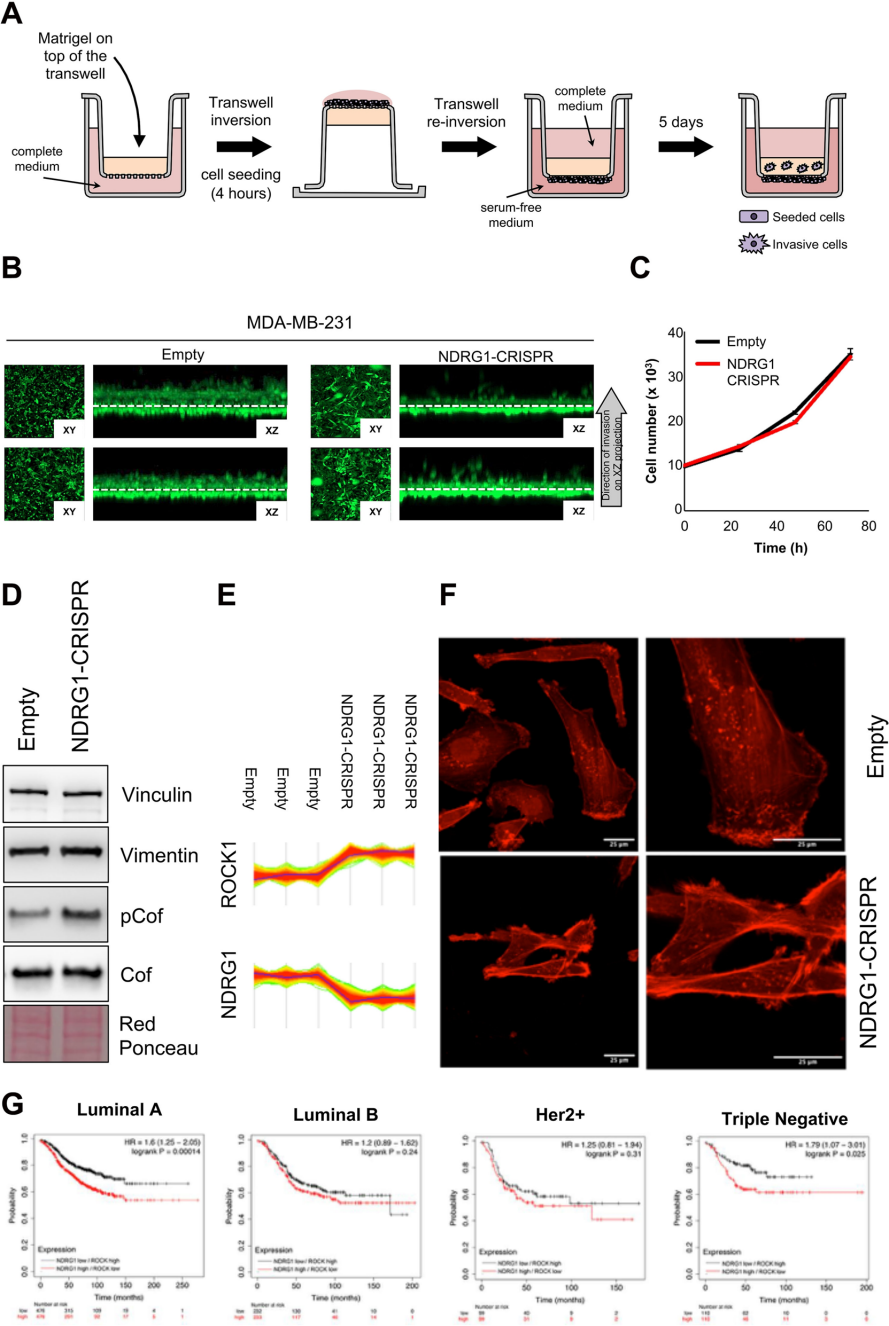
NDRG1 is required for breast cancer cell invasion in vitro. **A** Schematic representation of inverse Matrigel invasion test. **B** Inverse Matrigel invasion of MDA-MB-231 Empty and NDRG1-CRISPR cells. Green fluorescent cells result from live-cell staining with calcein-AM. The grey arrow indicates the direction of cell migration during the process of Matrigel invasion. **C** In vitro growth rates of MDA-MB-231 Empty and NDRG1-CRISPR cells. Empty CRISPR cells are in black; NDRG1 CRISPR cells are in red. Bars, SEM. **D** Western blotting analysis for Vinculin, Vimentin, phospho-Cofilin, and Cofilin of lysates obtained from MDA-MB-231 Empty and NDRG1-CRISPR cells. **E** The windows contain the expression profiles of NDRG1 and ROCK obtained after LC–MS/MS analysis. **F** Actin localization visualized using phalloidin staining of MDA-MB-231 Empty and NDRG1-CRISPR cells. **G** The Kaplan‒Meier Plotter website was used to investigate the relationship between NDRG1 low/ROCK high and NDRG1 high/ROCK low expression and survival probability in BC human samples

To characterize the functional consequences of NDRG1 knockdown at the proteome level, we analyzed Empty and NDRG1-CRISPR cells by LC–MS/MS (Suppl. MS/MS Data 4, Fig. 9C). Analysis of the differentially expressed proteins resulted in a heatmap which segregated the two samples in two main groups. NDRG1 was confirmed to be significantly inhibited in the CRISPR group. GO enrichment analysis of proteins that are significantly decreased (n = 523) in the NDRG1-CRISPR samples revealed overrepresentation of ‘‘Protein processing in endoplasmic reticulum (p = 1.2E−4)’’ and ‘‘Nucleocytoplasmic transport (p = 2.6E−3)’’ processes, whereas “Adherens junction (p = 6.3E−7) and “Regulation of actin cytoskeleton (p = 4.1E−4)” emerged as overrepresented among the proteins increased in NDRG1-CRISPR sample (n = 314) (Fig. S7A–D). The enrichment of these pathways in our dataset suggested functional consequences of NDRG1 modulation on ER protein processing, and cell migration and invasion. Analysis of different expressed genes was also performed between Empty and NDRG1-CRISPR cells by RNA-Seq. When filtering the data by log2FoldChange >|2| and adjusted p-value < 0.05, 509 genes were found to deregulated. Of these, 360 genes were downregulated in NDRG1-CRISPR samples. Pathway analysis of deregulated genes revealed an enrichment of processes associated with “Interferon alpha/beta signaling (p = 0.0005)”, “Post-translational protein phosphorylation (p = 1.1E−06)” and “Extracellular matrix organization (p = 0.0004)” (Fig. 9D). In the Extracellular matrix organization pathway, all genes show a concerted significant decrease in abundance in NDRG1-CRISPR compared to Empty samples (Fig. S8), potentially implicating NDRG1 as important for cell invasion. Moreover, none of the other NDRG family members (2, 3 and 4) change their expression after NDRG1 silencing (data not shown), confirming that the observed gene modulation is attributable only to NDRG1. Overall, these data underline a possible reduced capacity of NDRG1-CRISPR cells to invade the extracellular matrix as a consequence of the reduced expression of matrix-degrading enzymes and proteins involved in the regulation of the actin cytoskeleton. To demonstrate this, we analyzed the invasion of Empty (NDRG1-positive) and of NDRG1-CRISPR (NDRG1-negative) cells through inverted Matrigel invasion assays (Fig. 10A). As shown in Fig. 10B, we found that NDRG1 knockdown significantly reduced the invasion of MDA-MB-231 cells after 5d. As there was no difference in the cell proliferation of Empty and NDRG1-CRISPR cells (Fig. 10C), the migratory/invasive phenotype observed in our assay appeared to be independent from cell growth. The levels of proteins involved in cell migration were examined by western blotting including total cofilin protein (dephosphorylated and phospho-cofilin), vinculin, and vimentin. As shown in Fig. 10D, levels of phosphorylated (inactive) cofilin in NDRG1-CRISPR cells are significantly higher than in the controls. This is consistent with the proteomic data showing that the expression of ROCK is significantly increased in NDRG1-CRISPR cells (Fig. 10E). No differences were observed in the levels of vinculin and vimentin. As cofilin is a critical modulator of actin reorganization, we stained Empty and NDRG1-CRISPR cells with phalloidin to visualize actin organization. Confocal microscopy showed that the F-actin filaments are thin and long and converge toward the front of lamellipodium within the whole Empty and NDRG1-CRISPR cells. On the contrary, the F-actin filaments are loosely arranged near the lamellipodia of Empty cells compared to NDRG1-CRISPR (Fig. 10F). Inactivation of cofilin by serine phosphorylation may impact actin polymerization and lamellipodium formation in NDRG1-CRISPR cells.

Lastly, the prognostic significance of NDRG1/ROCK ratio was investigated using the KM plot analysis. According to KM data, we found that BC patients with higher expression of NDRG1 and lower expression of ROCK had poor survival outcomes compared to patients with lower expression of NDRG1 and higher expression of ROCK, in luminal A and TNBC subtypes (Fig. 10G).

## Discussion

TNBC is a heterogeneous BC subgroup with an aggressive clinical course. The identification and characterization of novel molecular pathways and targets are pivotal for the design of innovative therapeutic strategies. Increasing evidence indicates the oncogenic role of NDRG1 in different tumor types including TNBCs. In this study, we describe a different expression of NDRG1 in different BC tumor subtypes, as assessed by proteomics, RNA-Seq, and IHC, but also provide evidence of the upregulation of NDRG1 in BC tumor tissues compared to the normal counterpart in support for the possible oncogenic role of NDRG1. Our findings provided evidence to support this pathogenetic role and strengthened the prominent role for NDRG1 in TNBC tumors that are highly invasive. In fact, according to the molecular classification of TNBC performed by Lehmann BD and collaborators in 2011 [24], *NDRG1* is among the most up-regulated genes in basal-like 1 (BL1) and mesenchymal (M) tumors compared to the other subtypes. M tumors are enriched in pathways associated with cell motility and differentiation (i.e. Wnt and TGF-β). This is consistent with a recent study documenting that NDRG1 could have a pro-oncogenic function downstream of the TGF-β pathway [25] and this is also consistent with our experimental data demonstrating a reduced invasive ability of NDRG1-CRISPR cells compared to control cells. Our data offer some insight as to how NDRG1 regulates cell invasion. First, we identified a new molecular mechanism that drives the expression and activity of NDRG1 through the modulation of cofilin phosphorylation by ROCK. Previous studies have suggested that dephosphorylation and activation of cofilin facilitates actin reorganization and polymerization, a process that is directly related to BC metastasis [26, 27]. Here, we provide evidence for the functional connection between NDRG1 and ROCK. NDRG1 expression is inversely associated with ROCK expression in MDA-MB-231 cells. Down-regulation of NDRG1 significantly modulates ROCK expression and cofilin phosphorylation. Moreover, unbiased transcriptomic analysis of NDRG1-CRISPR cells compared to control cells revealed an under-representation of genes involved in the extracellular matrix organization pathway including integrin-β4 (ITGB4) and ADAM metallopeptidase domain 12 (ADAM12) that are associated with BC cell migration and invasion and poor prognosis in patients with TNBC [28, 29].

In our cohort of BC patients, NDRG1 expression was inversely related to the expression of ER, PgR, AR, and Her2 status and directly related to Ki67, showing a strong association with TNBC phenotype. The relationship with an aggressive phenotype was underlined by the survival analysis, where Kaplan–Meier curves showed a worse clinical outcome in the subgroup of TNBC with high NDRG1 expression. Further, in the BC group, patients with low TILs/high NDRG1 tumors had a worse DFS than the other subtypes considered. TILs are important prognostic and predictive biomarkers in BC and their high presence is a favorable prognostic factor in early-stage TNBCs [30]. The combination of low TILs presence and high NDRG1 expression could be a negative prognostic indicator of an unfavorable microenvironment. This is the first evidence of the interaction of NDRG1 with the tumor environment, highlighting a new role of this protein in cancer onset and progression. In agreement, data from spatial proteomics demonstrated a higher expression of NDRG1 in the tumor core and peripheral tumor. Many microenvironmental influences such as nutrient and oxygen availability are most prevalent in these tumor regions.

Moreover, in multivariate analysis, high NDRG1 expression and positive node status were independently associated with poorer DFS. In silico analysis underlined a different involvement of *NDRG1* expression according to different phenotypes. The differences between basal low and luminal high could be due to a more aggressive behavior of basal-like phenotype, without a direct involvement of NDRG1. Thus, the apparent inconsistency between gene and protein expression concerning patients’ outcomes could be explained by possible NDRG1 post-transcriptional modifications. Further, in silico analysis assumes that basal-like BCs are identical to TNBC, but TNBC and basal-like tumors are heterogeneous and the overlap is incomplete, by varying between 60 and 90% [31]. Interestingly, multivariate analysis indicated that NDRG1 is an independent prognostic factor for OS. These data are in agreement with those obtained in other studies demonstrating that NDRG1 and p-NDRG1 (Thr346) are associated with worse survival outcomes in TNBC [25]. Concerning the subcellular localization, we show by IHC in human BC tissues that NDRG1 is mostly localized in the cytoplasm and membrane. NDRG1 is localized to the nucleus only in tissue sections scored with 3 + staining intensity. This behavior emphasizes a NGRG1 dynamism associated with its oncogenic activity. DFS data concerning protein localization showed an interesting indicator of the possible dual role of NDRG1. In fact, when it is localized at the membrane level it is an indicator of a better prognosis. Its translocation to cytoplasmic and/or nuclear compartment is linked to a worse prognosis. Our results converge in the same direction in both the whole BC and the TNBC group, although in the latter the data do not have statistically significant differences, probably due to the small number of cases. However, the differential membrane/cytoplasmic expression of NDRG1 seems a new valuable prognostic biomarker for BC and in particular for TNBC patients. Notably, our results could provide a simple elucidation about the dual activity reported for this protein, in line with many Janus-faced proteins.

In contrast to the data regarding NDRG1, proteomic and RNA-seq data provide only partial indications of the other NDRG family members. In detail, tissue proteomic data show no alterations in the expression levels of the other isoforms. Conversely, proteomic analysis of BC cell lines demonstrates upregulation of both NDRG1 and NDRG3 in the TNBC model MDA-MB-231. This could lead to the hypothesis of a common regulatory mechanism, i.e. hypoxia [32], but does not provide information on the biological role of the isoform 3. The analysis of its expression levels in the NDRG1-CRISPR cell line shows that NDRG3 might not compensate for the absence of NDRG1, leading to the hypothesis of a specific and different biological role for these proteins. In vivo, data from Kim MC and collaborators demonstrated that NDRG3 is associated with aggressive phenotype and unfavorable outcomes in patients with invasive BC [33]. This underscores its clinical potential but further studies will be necessary to clarify its oncogenic role.

Regarding the overexpression of NDRG1 in vivo, several potential mechanisms should be considered. NDRG1 is overexpressed under stress conditions and proteomic data demonstrate an enrichment of stress-related biological processes in tumor tissues compared to healthy counterparts. This means that NDRG1 upregulation can be considered as a marker of the activation of specific biological processes that may cooperate in TNBC by promoting tumor progression and relapse [34]. Transcriptional and post-transcriptional regulatory mechanisms should be taken into account to explain NDRG1 overexpression. *NDRG1* is a target gene of transcriptional factors including YAP, XBP1, and SGK1 that are frequently up-regulated in TNBC [35–37]. In addition to the transcriptional regulation of *NDRG1*, data also support post-transcriptional mechanisms that regulate NDRG1 through the mTORC2 pathway. In fact, the phosphorylation of NDRG1 is considered a marker of pathway activation status [38]. Here, we demonstrate that different stimuli converge on PKC activation to up-regulate NDRG1 expression. These include iatrogenic stimuli but also metabolic conditions such as an overload of saturated fatty acids. Treatment of MDA-MB-231 cells with these stimuli and with the PKC activator, PMA, increases NDRG1 levels and phosphorylation. Our findings suggest that p53 may act as a positive regulator of *NDRG1* transcription. In fact, treatment with PMA or thapsigargin increases p53 expression with a concomitant increase of NDRG1 levels in MDA-MB-231 cells. Consistently, PMA was found to signal through a canonical pathway that induces the phosphorylation of MARCKS and Erk1/2. Interestingly, treatment with PMA reduced Cof phosphorylation levels suggesting that inactivation of the ROCK/Cof signaling pathway may be important in mediating NDRG1 upregulation. Indeed, treatment of cells with the ROCK inhibitor Y-27632 alone induces an increase of NDRG1 expression.

**Fig. 11:**
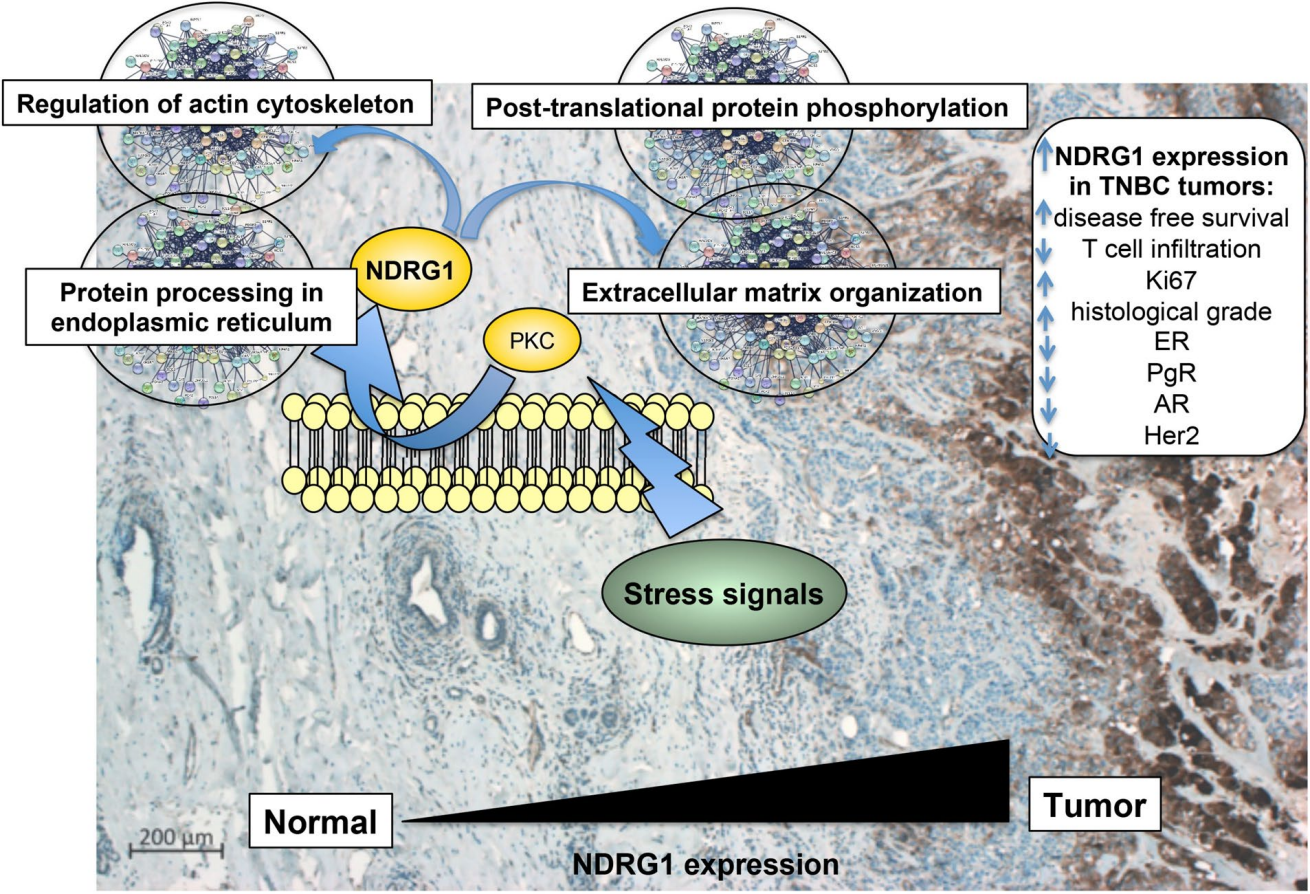
A schematic representation of the prognostic and molecular impact of NDRG1 in TNBCs. Tumor cell expression was found to be significantly increased compared to normal tissues and correlated with poor patient outcomes and molecular characteristics. Stress signals including chemotherapy or hostile environmental conditions converge to PKC to regulate NDRG1 expression through enhanced transcription which in turn regulates multiple pathways associated with endoplasmic reticulum and invasion

In conclusion, our data support the oncogenic role of NDRG1 and correlate its expression and localization with unfavorable clinical outcomes in BC patients. In detail, NDRG1 cellular localization correlates with poor prognosis, when the protein has a cytoplasmic localization Moreover, this study provides a detailed report of the role of NDRG1 in TNBCs and demonstrates that its prognostic significance is likely dependent on the modulation of cellular pathways associated with tumor response to stress and invasion, and the recruitment of immune cells (Fig. 11). Based on our results, NDRG1 expression falls under the regulation of PKC, and this is in line with the ability of PKC to promote BC aggressiveness.

## Supplementary Information

Additional file 1.

Additional file 2.

Additional file 3.

Additional file 4.

Additional file 5.

## Data Availability

All data analyzed during this study are included in in the main text or the supplementary materials. Mass spectrometry data have been deposited to the ProteomeXchange Consortium via the PRIDE partner repository with the dataset identifier PXD045164. Correspondance and requests for RNA Seq data should be addressed to s.desumma@oncologico.bari.it.
