# Supplementary material for: Unraveling the protein kinase C/NDRG1 signaling network in breast cancer
Source: Cell Biosci. 2024 Dec 30;14:156. doi: 10.1186/s13578-024-01336-z (PMC11686873; doi:10.1186/s13578-024-01336-z)
Supplement: Supplementary file 1 — Additional file 1. [file 13578_2024_1336_MOESM1_ESM.docx]

**Supplemental Information**

**Unraveling the Protein Kinase C/NDRG1 Signaling Network in Breast Cancer**

**Saponaro C^1^, Damato M^2^, Stanca E^2^, Aboulouard S^3^, Zito FA^1^, De Summa S^4^, Traversa D^4^, Schirosi L^1^, Bravaccini S^5^, Pirini F^6^, Fonzi E^7^, Tebaldi M^7^, Puccetti M^8^, Gaballo A^9^, Pantalone L^10,11^, Ronci M^10,11^, Magnani L^12,13,14^, Sergi D^15^, Tinelli A^16^, Tacconi S^17^, Siculella L^2^, Giudetti AM^18^, Fournier I^3^, Salzet M^3^, Trerotola M^10,11#*^, Vergara D^18#*^**

^1^Pathology Department, IRCCS Istituto Tumori "Giovanni Paolo II", 70124 Bari, Italy

^2^Department of Experimental Medicine, University of Salento, Lecce, Italy

^3^Lille University, Inserm, CHU Lille, U1192, Laboratoire Protéomique, Réponse Inflammatoire et Spectrométrie de Masse (PRISM), Lille, France

^4^Molecular Diagnostics and Pharmacogenetics Unit, IRCCS Istituto Tumori "Giovanni Paolo II", 70124 Bari, Italy

^5^Faculty of Medicine and Surgery, "Kore" University of Enna, 94100 Enna, Italy

^6^Biosciences Laboratory, IRCCS Istituto Romagnolo per lo Studio dei Tumori (IRST) "Dino Amadori", Meldola, Italy

^7^Unit of Biostatistics and Clinical Trials, IRCCS Istituto Romagnolo per lo Studio dei Tumori (IRST) "Dino Amadori", Meldola, Italy

^8^Azienda Unità Sanitaria Locale di Imola, Imola, Italy

**^9^**CNR Nanotec, Institute of Nanotechnology, Via Monteroni, 73100 Lecce, Italy

^10^Laboratory of Cancer Pathology, Center for Advanced Studies and Technology (CAST), "G. d'Annunzio" University of Chieti-Pescara, Chieti, Italy

^11^Department of Medical, Oral and Biotechnological Sciences, "G. d'Annunzio" University of Chieti-Pescara, Chieti, Italy

^12^The Breast Cancer Now Toby Robins Research Centre, The Institute of Cancer Research, London, United Kingdom

^13^Department of Surgery and Cancer, Imperial College London, London, United Kingdom

^14^Department of Oncology and Haemato-Oncology, Università degli Studi di Milano, Milan, Italy

^15^Department of Radiology - V. Fazzi Hospital, 73100 Lecce, Italy

^16^Department of Obstetrics and Gynecology and CERICSAL (CEntro di RIcerca Clinico SALentino), “Veris delli Ponti Hospital”, Scorrano, 73020, Scorrano (Lecce), Italy

^17^Department of Biology and Biotechnology “Charles Darwin”, Sapienza University of Rome, P.le Aldo Moro 5, 00185, Rome, Italy

^18^Department of Biological and Environmental Sciences and Technologies (DiSTeBA), University of Salento, Lecce, Italy

# co-last authors

*** Correspondence:**Corresponding Authors
[daniele.vergara@unisalento.it](mailto:daniele.vergara@unisalento.it)

marco.trerotola@unich.it

**Keywords: NDRG1, Breast Cancer, TNBC, PKC**

**Supplemental Tables**

**Table S1.** Clinical and pathologic characteristics of the breast cancer cohort (n=211).

|  | **N. (%)** |
| --- | --- |
| **Age (years):** median value (range 29-80) | 53 |
| ≤53 | 108 (51) |
| >53 | 103 (49) |
| **Histotype** |  |
| IDC | 190 (90) |
| ILC | 10 (5) |
| Other | 10 (5) |
| unknown | 1 |
| **Tumor size (cm)** |  |
| ≤2.0 | 118 (56,5) |
| >2.0 | 91 (43,5) |
| Unknown | 2 |
| **Node** |  |
| Negative | 123 (59) |
| Positive | 85 (41) |
| unknown | 3 |
| **Grade** |  |
| 1 | 10 (5) |
| 2 | 95 (45) |
| 3 | 105 (50) |
| unknown | 1 |
| **ER (%)** |  |
| <1 | 92 (44) |
| ≥1 | 119 (56) |
| unknown | / |
| **PgR (%)** |  |
| <1 | 102 (48) |
| ≥1 | 109 (52) |
| **AR (%)** |  |
| <1 | 105 |
| ≥1 | 87 |
| unknown | 20 |
| **Ki67 (%)** |  |
| <14 | 63 (30) |
| ≥14 | 146 (70) |
| unknown | 2 |
| **HER2** |  |
| Negative | 195 (92) |
| Positive | 16 (8) |
| unknown | / |
| **Molecular Subtype** |  |
| Luminal | 140 (66) |
| TNBC | 71 (34) |
| **NDRG1** |  |
| low | 106 (50) |
| high | 105 (50) |
| unknown | / |
| **TILs** | 40 |
| low | 129 (75%) |
| high | 43 (25%) |
| unknown |  |
| IDC: Invasive ductal carcinoma; ILC: Invasive lobular carcinoma; ER: Estrogen receptor; PgR: Progesterone receptor; AR: Androgen receptor; HER2: Human epidermal growth factor  receptor 2; NDRG1: N-Myc downstream- regulated 1; TILs: tumour-infiltrating lymphocyte | |

**Table S2**. Gene Ontology analysis performed by STRING version 12.0.

| **Reactome pathway** | | | | |
| --- | --- | --- | --- | --- |
| **pathway** | **description** | **count in network** | **strength** | **false discovery rate** |
| HSA-8953854 | Metabolism of RNA | 106 of 705 | 0.86 | 4.84e-53 |
| HSA-392499 | Metabolism of proteins | 156 of 1917 | 0.6 | 5.36e-49 |
| HSA-5663205 | Infectious disease | 101 of 917 | 0.73 | 1.54e-39 |
| HSA-72766 | Translation | 63 of 290 | 1.02 | 1.81e-38 |
| HSA-2262752 | Cellular responses to stress | 89 of 747 | 0.76 | 6.65e-37 |
| **KEGG pathway** | | | | |
| **pathway** | **description** | **count in network** | **strength** | **false discovery rate** |
| hsa01100 | Metabolic pathways | 53 of 1345 | 0.25 | 0.00051 |
| Hsa04141 | Protein processing in endoplasmic reticulum | 31 of 163 | 0.97 | 5.85e-17 |
| **Biological process** | | | | |
| **GO term** | **description** | **count in network** | **strength** | **false discovery rate** |
| GO:0033554 | Cellular response to stress | 68 of 1572 | 0.32 | 9.30e-07 |
| GO:0006950 | Response to stress | 112 of 3358 | 0.21 | 9.15e-06 |
| GO:0080135 | Regulation of cellular response to stress | 38 of 712 | 0.41 | 1.71e-05 |
| GO:0080134 | Regulation of response to stress | 58 of 1373 | 0.31 | 2.21e-05 |
| GO:0034976 | Response to endoplasmic reticulum stress | 19 of 223 | 0.62 | 3.92e-05 |
| GO:1902882 | Regulation of response to oxidative stress | 8 of 93 | 0.62 | 0.0368 |

**Table S3.** Relationship between NDRG1 and clinicopathological features.

|  | **NDRG1 expression** | | |
| --- | --- | --- | --- |
|  | **low** | **high** |  |
|  | **N (%)** | **N (%)** | **p** |
| **Age** | | | |
| ≤53 | 50 (47) | 58 (55) |  |
| >53 | 56 (53) | 47 (45) | 0.24 |
| **Histotype** | | | |
| IDC | 89 (85) | 101(97) | **0.01** |
| ILC | 9 (9) | 1 (0.9) |  |
| Other | 7 (6) | 3 (2.1) |  |
| **T. size (cm)** | | | |
| ≤2.0 | 64 (61) | 54 (52) | 0.18 |
| >2.0 | 41 (39) | 50 (48) |  |
| **Node** | | | |
| Negative | 63 (60) | 60 (58) | 0.79 |
| Positive | 42 (40) | 43 (41) |  |
| **Grade** | | | |
| 1-2 | 70 (67) | 35 (34) |  |
| 3 | 35 (33) | 69 (66) | **<0.0001** |
| **ER (%)** | | | |
| <1 | 18 (17) | 74 (70) | **<0.0001** |
| ≥1 | 88 (83) | 31 (30) |  |
| **PgR (%)** | | | |
| <1 | 25 (24) | 77 (73) | **<0.0001** |
| ≥1 | 81 (76) | 28 (27) |  |
| **AR (%)** | | | |
| <1 | 46 (46) | 59 (63) | **0.0182** |
| ≥1 | 53 (53) | 34 (37) |  |
| **Ki67 (%)** | | | |
| <14 | 50 (47) | 13 (13) |  |
| ≥14 | 56 (53) | 90 (87) | **<0.0001** |
| **HER2** | | | |
| Negative | 93 (88) | 102 (97) | **0.009** |
| Positive | 13 (12) | 3 (3) |  |
| **TILs** |  |  |  |
| low (<50%) | 69 (84%) | 60 (67%) | **0.008** |
| high (≥50%) | 13 (16%) | 30 (33%) |  |
| p-value of Chi-squared test for the independence of categorical variables. Bold values indicate significanceDC: Invasive ductal carcinoma; ILC: Invasive lobular carcinoma; ER: Estrogen receptor; PgR: Progesterone receptor; AR: Androgen receptor; HER2: Human epidermal growth factor receptor 2; NDRG1: N-Myc downstream- regulated 1; TILs: tumour-infiltrating lymphocyte | | | |

**Table S4.** Univariate analysis of DFS (disease-free survival).

| **Characteristic** | **N** | **HR^1^** | **95% CI^1^** | **p-value** |
| --- | --- | --- | --- | --- |
| **Age** | 193 |  |  |  |
| 1 |  | — | — |  |
| 2 |  | 0.59 | 0.28, 1.22 | 0.15 |
| **Node** | 192 |  |  |  |
| Negative |  | — | — |  |
| Positive |  | 2.23 | 1.11, 4.46 | 0.023 |
| **NDRG1** | 193 |  |  |  |
| Low |  | — | — |  |
| High |  | 2.59 | 1.20, 5.59 | 0.016 |
| **Molecular subtype** | 193 |  |  |  |
| Luminal |  | — | — |  |
| TNBC |  | 2.08 | 1.04, 4.16 | 0.039 |
| ^1^HR = Hazard Ratio, CI = Confidence Interval | | | | |

**Table S5.** Multivariate analysis of DFS (disease-free survival).


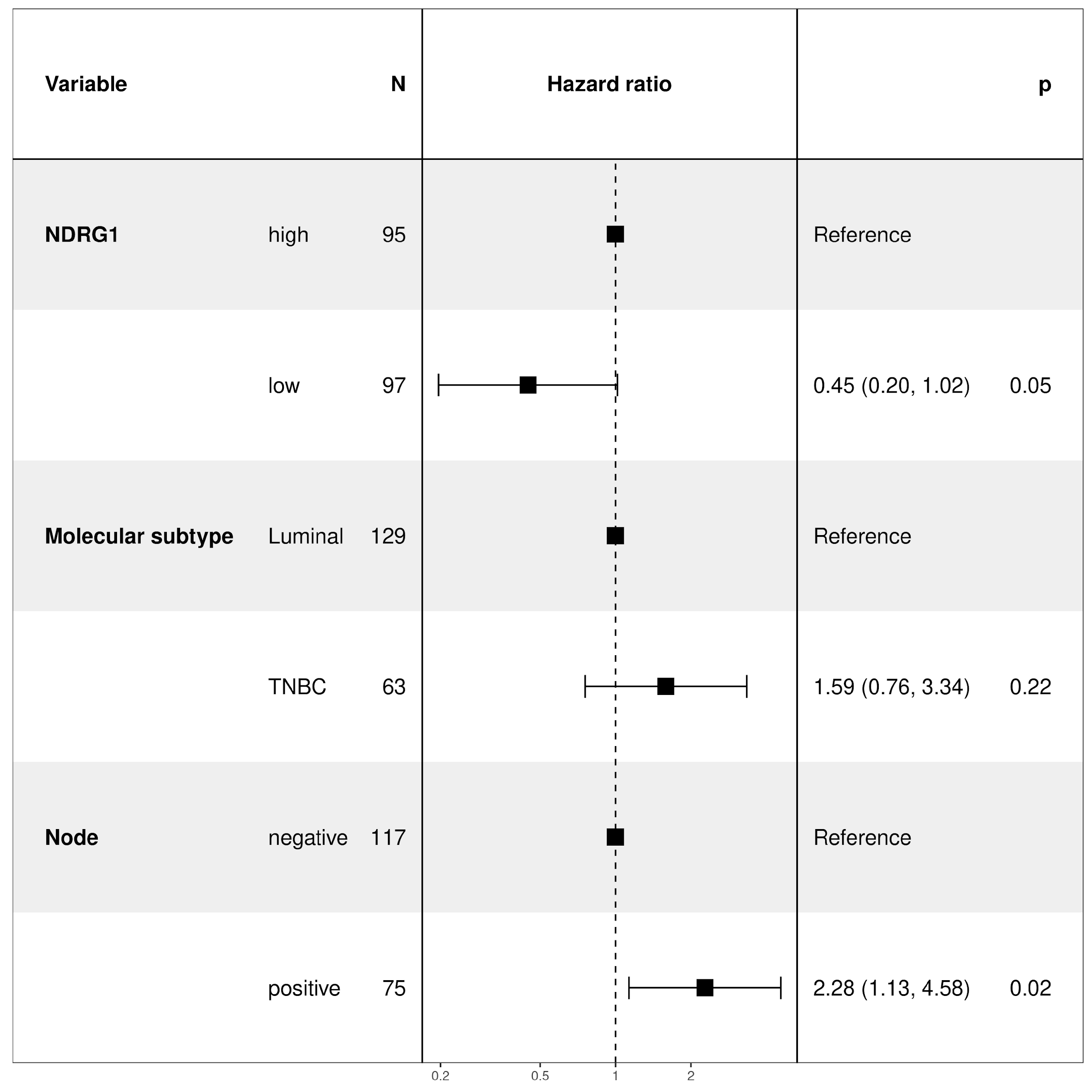


**Supplemental Experimental procedures**

**Human tissues analysis**

**Case series**

Twelve breast cancer patients (6 triple negative, 3 luminal A, and 3 luminal B/Her2 positive) were enrolled at the Hospital Santa Maria della Scaletta, Imola (Italy). The study was conducted in accordance with ethical standards, the Declaration of Helsinki, and national and international guidelines, and was approved by local ethics committee (CE AVEC-protocol number 10547). All the patients enrolled in the study have signed an informed consent for the use of the results for research purposes. The inclusion criterion was the availability of formalin-fixed paraffin-embedded (FFPE) primary tumour tissue not older than 2017 to limit the degradation of biological material. One section of 4 μm of all samples were stained by hematoxylin and eosin and reviewed by a dedicated pathologist for the selection of tumour and healthy areas. One section of 10 μm was used for the mass spectrometry analysis and two 10 μm freshly cutted sections were used for RNA extraction. Two hundred and eleven retrospective, non-consecutive primary invasive BCs were enrolled at the Istituto Tumori “Giovanni Paolo II” of Bari (Italy). The patients were selected on the availability of biological material and their clinical follow-up. Patients were eligible if they had a histological diagnosis of invasive BC of any size and no evidence of metastatic disease at diagnosis. The study was approved by the Ethics Committee of the Istituto Tumori “Giovanni Paolo II” (no. 1310/CE of July 2023). The median age was 53 years. The tumor, node, metastasis (TNM) classification, histological grade, androgen receptor (AR), estrogen receptor (ER) and progesterone receptor (PgR) status, proliferative activity (Ki67) expression, and human epidermal growth factor receptor 2 (HER2) status were provided by the pathology departments of the two institutes, respectively. Tumors with ER or PgR expression were classified as positive when nuclear staining was found in ≥ 1% [WHO 2019]. The AR status was defined as positive when tumor cells showed positive staining ≥ 1%. Ki67 nuclear staining was used to assess the proliferative activity, with a cut-off value of ≥14% of positive cells to indicate a highly proliferative tumor [WHO 2019]. Cases Her2 scored as 0 and 1+ were classified as negative. HER2 was considered to be positive if immunostaining was 3+ or 2+ with gene amplification by FISH, according to the 2018 ASCO/CAP guidelines for BC [1]. The molecular subtype was assigned according to the WHO 2019 guidelines for BC. In specific, the cases were considered Luminal A with ER ≥1%, PgR ≥1% and Ki67 <14%; Luminal B with ER ≥1%, PgR ≥1%, Ki67 ≥14%; Triple Negative Breast Cancers (TNBCs) with ER <1%, PR <1% and HER2 negative. We also considered the presence (low/high) of tumour-infiltrating lymphocytes (TILs). Table S1 summarizes the clinicopathological characteristics of the entire cohort.

**Immunohistochemistry**

Consecutive sections of 4-µm thickness were cut from FFPE samples and stained with an indirect immunoperoxidase method using the BenchMark XT automated staining instrument (Ventana Medical Systems). Antigen retrieval was made with Cell Conditioning solution 1 at 95°C. The slides were then incubated at 37°C for 1 h with Anti-NDRG1 antibody rabbit polyclonal (Sigma-Aldrich, Merck KGaA) diluted 1:100 in phosphate-buffered saline/bovine serum albumin (PBS/BSA) 1% and 36 minutes with anti-human androgen receptor (AR) antibody mouse monoclonal (clone AR441, Dako Agilent) diluted 1:50 in antibody diluent (Ventana-Roche). The UltraView DAB IHC Detection Kit (Ventana Medical Systems) was used to detect protein expression. Finally, tissues were counterstained with hematoxylin and a bluing reagent for 8 min and 4 min respectively, then dehydrated and mounted. Positive and negative controls were included in each staining run as indicated in the datasheet of each antibody. All of the antibodies used in this study were validated in the pre-analytic phase to guarantee a satisfactory level of reproducibility and accuracy. All the solutions were from Ventana Medical Systems unless otherwise specified.

**Immunohistochemical Assessment**

Membrane and cytoplasmic expression of NDRG1 was considered. Sections were examined under a light microscope (Zeiss, Oberkochen, Germany) and a H-score was assessed. The H-score was calculated by the product of the percentage of positive cells (0%–100%) and the intensity of the staining (1=weak, 2=moderate and 3=strong) [2]. The median (value of 10) was used as a cutoff, wherein 106 patients were grouped as NDRG1-low (≤10) and 105 as NDRG1-high (>10). The examination of AR expression was assessed based on nuclear staining intensity and 1% was used 1 as a cut-off value (negative <1%; and positive ≥1%) [3]. All stained specimens were assessed independently by two observers who were blinded to the clinicopathological data. Three distinct visual fields were selected to evaluate the slides using x400 magnification. Discordant scores were reviewed and resolved by discussion.

**Quantification of tumor-infiltrating lymphocytes**

Tumor-infiltrating lymphocytes (TILs) were assessed in hematoxylin and eosin-stained FFPE sections of tumor tissue (4–5 μm, magnification ×200) from the surgical specimen, according to TILs Working Group recommendations [4]. TILs were scored in the stromal compartment as the percentage of all mononuclear cells in the area of stromal tissue as previously reported [5]. Only lymphocytes inside the borders of the invasive tumor, including the invasive margins, were evaluated; immune infiltration outside the tumor border, necrosis, and lymph nodes were excluded. Tumors with a stromal TILs score of ≥50% were considered lymphocyte-predominant BC.

**Immunofluorescence**

Formalin-fixed and paraffin embedded tissue serial sections of 3 μm in thickness were deparaffinized with xylene, and rehydrated in an ethanol series. Antigen retrieval was carried out immersing slide in a 0.01 M Tris-EDTA buffer, 0.05% Tween 20 (pH 8.9) at 98 °C for 30 minutes, then tissues were permeabilized with 0.1% Triton X100-Phosphate Buffered Saline for 10 minutes, blocked with 2% Goat Serum in 1X PBS for 45 minutes and incubated overnight at 4 °C in a humidified chamber with a mouse monoclonal Pan Cytokeratin_ Alexa Fluor 488 (clone AE1/AE3, dilution 1:100; Thermo Fisher Scientific Inc.), together with a rabbit polyclonal anti-NDRG1 (Sigma-Aldrich, Merck KGaA; dilution 1:100) in 0.1% Goat Serum in 1X PBS_0.05%Tween20. The anti-rabbit Alexa Fluor 568 immunoglobulinG secondary conjugated antibody (1:2000 dilution;Molecular Probes Inc., Eugene, OR, USA) was incubated in 0.1% Goat Serum in 1X PBS_0.05%Tween20, at room temperature for 1 h. Then the slides were treated with Vector True View Autofluorescence Quenching kit for 2 minutes, to reduce tissue autofluorescence, and mounted with VECTASHIELD Vibrance antifade mounting medium with DAPI (Vector Laboratories, Inc;Burlingame, CA, United States). Positive control slides that were run simultaneously were used for assessing the quality of immunoreactivity. For negative controls, slide sections that were immunopositive were treated with 0.1% Goat Serum instead of the primary antibody, and no reactivity was observed in any of these controls. Images were obtained on an Axion Image 2 upright microscope (Zeiss, Oberkochen, Germany) with an Axiocam 512 color camera.

**RNA extraction and sequencing of human tissues**

RNA extraction was performed using two 10 μm consecutive sections or more in case of small areas. Tumor tissues were selected on the base of the H&E and separately extracted by the Allprep DNA/RNA FFPE kit (Qiagen). Samples were quantified using a Qubit fluorometer (Thermo Fisher Scientific). The quality and integrity of the RNA were evaluated by a bioanalyzer. Libraries preparation was performed using NEBNext Ultra II Library Prep kit for Illumina with rRNA depletion. Libraries were sequenced on NextSeq500 (Illumina). Reads count was performed with kallisto (v0.43.1) [6] against Ensembl version 94 of the human transcriptome (https://github.com/pachterlab/kallisto-transcriptome-indices/releases). Differential gene expression analysis between TNBC and Luminal BC samples was performed on raw read counts with DESeq2 (v1.22.1) [7].

**Follow-up and Statistical Analysis**

Disease Free Survival (DFS) was defined as the time from the date of surgery to the date of first relapse or progression of disease or to the date of a second invasive BC/secondary primary cancer and/or death without evidence of BC or to the date of the last follow-up. Overall Survival (OS) was defined as the time between the date of surgery and the date of death from any cause or the date of the last follow-up. Time-to-event variables were estimated using the Kaplan-Meier method and comparisons between curves were done using the Log-rank test. To identify the prognostic factors for DFS and OS, univariate and multivariate Cox regression models were used to estimate hazard ratios (HR) and their 95% confidence intervals (95% CI). The R (version 4.2.1) packages ‘survival_3.4-0’, and ‘survminer_0.4.9’ were used to perform survival analyses. Kaplan-Meier curves and multivariate Cox results have been depicted through the R packages ‘ggplot2_3.3.6’ and ‘forestmodel_0.6.2’ The association of baseline factors and protein expressions was evaluated with the Chi-square test. The NDRG1 expression was evaluated using the Mann-Whitney test. Statistical analyses were performed using the Prism version 8.00 software package (Graph-Pad Software). p<0.05 was considered to be statistically significant (*p < 0.05, **p < 0.01, and ***p < 0.001). An *in silico* cohort has been set up to retrieve, through *TCGAbiolinks* [8], RNAseq and clinical data from the TCGA-BRCA cohort. Molecular subtype data were retrieved through the *TCGAbioquery_subtype* function, selecting PAM50 stratification. RNAseq STAR counts were normalized by variance stabilizing transformation embedded in the DESeq2 R package [7].

**Mass Spectrometry analysis**

**Dewaxing and antigen retrieval**

Each tissue was deparaffinized by soaking in xylene for 5 min, 2 times. The tissues were rehydrated for 5 min using each successive bath of decreasing degree of ethanol (2×95%, 1×30%) and two baths of 10 mM NH_4_HCO_3_ buffer. Then, antigen retrieval was performed in 20 mM Tris buffer pH9 for 30 min at 90°C, followed by two baths of 10 mM NH_4_HCO_3_ for 2 min. The slides were dried under vacuum at room temperature before the digestion step.

**Enzymatic digestion and localized extraction of digested proteins on tissue**

Digestion was performed by a chemical inkjet printer (CHIP-100, Shimadzu, Kyoto, Japan), using a trypsin solution (40 μg/mL, 50 mM NH_4_HCO_3_ buffer). The deposit was carried out for 3 h, on regions defined at 1mm^2^. With 400 cycles and 57.6 nl per spot, a total of 1 μg was deposited. To stop the digestion, 0.1% trifluoroacetic acid (TFA) was deposited for 25 cycles. The slides were then dried under vacuum before extracting the digested proteins.

After digestion, each spot content was collected by liquid microjunction using the TriVersa Nanomate (Advion Biosciences Inc.) using liquid extraction and surface analysis (LESA) parameters. Three different extraction buffers were prepared and were composed of 0.1% TFA (buffer 1), acetonitrile (ACN)/0.1% TFA (8:2, v/v) (buffer 2) and MeOH/0.1% TFA (7:3, v/v) (buffer 3). A complete LESA sequence performs 2 cycles of extractions for each buffer. The first step in a cycle was to aspirate 2 μl of solvent into a tip, 0.6 μl was dropped onto the tissue to create a liquid micro junction with 10 aspiration-dispensing cycles to perform the extraction, and the solution extract was collected in 0.2 ml low binding tubes. All collection tubes were dried under vacuum by the SpeedVac concentrator (SPD131DDA-230, Thermo ScientificTM), desalted using a ZipTip C-18 (Merck Millipore), and eluted with a solution of ACN/0.1% TFA (7:3, v/v). Samples were dried with SpeedVac and resuspended in 20 µL ACN/0.1% formic acid (0.2:9.8, v/v) and 6 µl injected into the nanoLC-MS/MS.

**Sample preparation and mass spectrometry analysis**

Whole protein extraction was carried out with the Illustra TriplePrep kit (GE Healthcare) according to the manufacturer’s protocol. Subsequently, almost 20 μg of protein extract were processed according to the filter-aided sample preparation (FASP) protocol. Protein extract was dissolved and denatured in 8M urea in 0.1 M Tris/HCl pH 8.5, reduced by an equivalent volume of 0.1M dithiothreitol (DTT) solution, and heated at 56°C for 40 minutes. The samples were loaded in an Amicon Ultra - 0.5 centrifugal filter device (Merck Millipore) with a molecular weight cut-off of 10kDa, washed three times with 8M urea, and centrifuged at 14,000xg for 30 min. This procedure was followed by the alkylation of the sample with 0.05M of iodoacetamide solution for 20 min in the dark and centrifugation at 14,000xg for 30 minutes. After three washes with 8M urea and three washes with 0.05M NH4HCO3, the protein samples were digested by 20μg/mL trypsin solution overnight at 37°C. Each passage was followed by a centrifugation at 14,000xg for 30 min. Peptides were collected by centrifugation followed by an additional wash with 0.5M NaCl. Finally, the peptide mixture was acidified by 0.1% TFA solution, desalted-concentrated on C-18 ZipTip pipette tips (Merck Millipore), eluted in 80% ACN and dried under vacuum. To reconstitute the mixture, the sample was then resuspended in 20 μL of ACN/H_2_O (FA 0.1%) (98:2, v/v).

**Mass spectrometry analysis and database searching**

The mass spectrometry analysis on the peptides was gained in reverse phase, using a chromatography system equipped with a pre-column (Acclaim PepMap 75 μm ID x 2 cm, 3 µm, Thermo Scientific) to pre-concentrate the peptides, and an analytical column (Acclaim PepMap RSLC 75 µm ID x 50 cm, 2 µm, Thermo Scientific), used for their separation. Elution was carried out using a 2h gradient of ACN/0.1% TFA starting from 5% to 30% for 120 min at a flow rate of 300 nL/min. The chromatographic system was coupled with a Q-Exactive Orbitrap mass spectrometer (Thermo Scientific) containing a nano-electrospray ionization source. The analyzer was set with a resolution of 70,000 FWHM, a m/z mass range between 300-1600, an automatic gain control (AGC) of 3E6 ions was used for full Mass Spectrometry (MS) scan and a maximum injection time of 120 ms. The minimum charge status of +2 was retained until +7, going to exclude the unassigned load states, the +1 and > +8 charges. The MS/MS analysis was carried out analysing the most intense ions within the primary MS study (top 10). The parameters of the MS/MS fragmentation were set at 17,500 FWHM, an m/z range between 200-2000, an AGC of 5E4 ions and a maximum time injection of 60 ms. MaxQuant proteomics software (version 1.6.3.4) was used to analyze MS/MS raw files in order to match peptide sequences in proteomes databases of Homo sapiens (Uniprot, released May 2019, 20406 entries) using the Andromeda algorithm [9-11]. The false discovery rate was set to 1% for peptides and protein identification and a minimum of 2 peptides per protein, with at least 1 unique, was defined. Label-free quantification of proteins was conducted using the MaxLFQ algorithm. Statistical analysis was performed with the Perseus software (version 1.6.10.43) [10]. Proteins that were identified in the decoy reverse database, potential contaminants and only identified by site modification were not considered for data analysis. Matrix data was transformed using the log2(x). Data were further filtered to make sure that identified proteins showed expression in at least 70% of the biological samples for each considered condition. Missing values were replaced assuming a normal distribution, through the imputation function (down shift = 1.8; width = 0.3). Two sample test (T-test) or multiple sample test (ANOVA) were performed (p-value = 0.01 or 0.05) in order to determine statistically significant differential protein expression. For hierarchical clustering, LFQ intensities were first z-scored and clustered using Euclidean as a distance measure for column and row clustering. Mass spectrometry data have been deposited to the ProteomeXchange Consortium [12] via the PRIDE partner [13] repository with the dataset identifier PXD045164.

**Cell culture analysis**

**Cell culture and treatments**

MDA-MB-231, MCF-7, MDA-361, T47D, Hs 578T, Huh7, HCT-15, HCT-116 cells were maintained in Dulbecco’s Modified Eagle’s Medium (DMEM) (4500 mg/L glucose, Sigma D5671) supplemented with 10% Fetal Bovine Serum (FBS, F9665 Sigma) (or FBS lipid-depleted, Biowest), 100 U/mL penicillin, 100 µg/mL streptomycin, and 2 mM glutamine at 37°C in an atmosphere of 5% CO_2_. To induce PKC activation, cells were stimulated with PMA (Santa Cruz) at the concentration of 100 nM, for 24 h. For cell treatments with fatty acids, 8 mM palmitic and oleic acids were prepared in bovine serum albumin (BSA) solution. Fatty acids were added to the cells for 24h at a final concentration of 200 uM. Control cells were treated with BSA alone. For inducing ER stress, thapsigargin was utilized at 1 μM for 24 h for 24 h. For inhibiting PI3K, LY294002 was utilized at 10 μM for 24 h. For inhibiting PKC, Ro 318220 was utilized at 1 μM for 24 h. The receptor tyrosine kinase inhibitors Vandetanib and Crizotinib were utilized at 10 μM for 24 h.

**Cell proliferation assay**

Cells were seeded at 104 cells/well in triplicate wells per data point using 96-well plates. Cell numbers were quantified every 24 h by staining with crystal violet. Briefly, cells were washed with PBS and fixed with PFA for 15 min at RT. Afterwards, 100 μL of 0.1% crystal violet in 50% methanol was added to each well, following by overnight incubation at RT. The wells were then washed for five times with water, and the crystal violet bound to the cells was solubilized with acetic acid. Absorbance was read at 550 nm, and the corresponding cell number was then calculated for each time point and each well. Data points are expressed as mean ± SEM.

**Confocal microscopy analysis**

For confocal microscopy analysis, cells were grown onto glass coverslips at 3 x 104 cells/ml in 6-well plates overnight. Cells were then fixed with 3.8% PFA, washed twice with PBS, and incubated with 0.1% Triton X-100 to permeabilize the cellular membrane. Cytoskeleton F actin filaments were stained with phalloidin–tetramethylrhodamine B isothiocyanate (TRITC), according to the manufacturer’s protocol (P1951, Sigma). After staining, images were acquired by using a CLSM Zeiss LSM700.

**Western blotting analysis**

Cell lysates were extracted in RIPA buffer (Cell Signaling) and quantified by the BRADFORD method (Bio-RAD). Twenty-five μg of proteins were mixed 1:1 with Laemmli buffer (Sigma) boiled for 5 min, separated by 12% SDS-PAGE, and transferred to the Hybond ECL nitrocellulose membrane (GE Healthcare). Subsequently, membranes were blocked for 1h in Blotto A (Santa Cruz) at room temperature, and incubated for 1-2 h at room temperature with a primary antibody diluted in Blotto A. After two washes of 10 minutes with tris-buffered saline tween (TBST) solution (10 mM Tris, pH 8.0, 150 mM NaCl, 0.5% Tween 20), membranes were incubated with horseradish peroxidase (HRP)-conjugated secondary antibodies for 2 h at room temperature. Membranes were washed twice for 5 min with TBST and then imaged using the ChemiDoc MP Imaging System (Bio-RAD). Membranes were probed with the following antibodies (1:1000 dilution): E-cadherin (#14472, Cell Signaling), Cofilin (sc-33779, Santa Cruz), phospho-Cofilin (Ser3) (sc-365882, Santa Cruz), Erk1/2 (#4695, Cell Signaling), phospho-Erk1/2 (Thr202/Tyr204) (#4370, Cell Signaling), BiP/GRP78 (#3177, Cell Signaling), NDRG1 (#5196, Cell Signaling), NDRG1 (HPA006881, Sigma-Aldrich), phospho-NDRG1 (ab124713, Abcam), NDRG2 (sc-376202, Santa Cruz), NDRG3 (sc-514561, Santa Cruz), NDRG4 (sc-514144, Santa Cruz), phospho-MARCKS (Ser167/170) (#8722, Cell Signaling), phospho-PKC Substrate Motif [(R/K)XPSX(R/K)] MultiMabTM (#6967, Cell Signaling), PKCα (#59754, Cell Signaling), PKCε (#2683, Cell Signaling), SGK1 (ab43606, Abcam), Vimentin (#5741, Cell Signaling), Vinculin (sc-73614, Santa Cruz), p38 (#9212, Cell Signaling), p53 (sc-126, Santa Cruz), YAP (sc-101199, Santa Cruz). Secondary antibodies (HRP-conjugated) from Santa Cruz Biotechnology (1:2000 dilution) (goat anti-mouse IgG-HRP, sc-2005; goat anti-rabbit IgG-HRP, sc-2004), or from Cell Signaling (1:2000 dilution) (anti-rabbit IgG, HRP-linked Antibody #7074, anti-mouse IgG, HRP-linked Antibody #7076) were used.

**RNA extraction and Real-time PCR**

Total RNA was extracted from cells grown in a 6-well plate using the Trizol (Sigma) following the manufacturer’s protocol. The reverse transcriptase reaction (20 μL) was carried out using 1 μg of total RNA iScript cDNA synthesis kit (Bio-RAD) according to the manufacturer’s protocol. Quantitative gene expression analysis was performed in a CFX Connect Real-time System (Bio-RAD) using iTaq Universal SYBR Green Supermix (Bio-RAD). The employed primer sequences were listed in the followings: *NDRG1* (NM_001135242.2), F: 5’-AAGTGGTCCACACCTACCG-3’, R: 5’-GGCATTGGTCGCTCAATCTC-3’ (160 base pairs); *NDRG3* (NM_022477.4), F: 5’-AGGTAGTTCAGCCTGGGAAG-3’, R: 5’-TTGAGTGGGTTCGTGATCGG-3’ (111 bp); *NDRG4* (NM_001130487.2) F: 5’-GCTGGTGAACATCGACCCC-3’, R: 5’-AGTGCTAGTTAGGCCGGAG-3’ (76 bp); *Rlpl0* (NM_001697.2), F: 5’-TCGACAATGGCAGCATCTAC-3’, R: 5’-ATCCGTCTCCACAGACAAGG-3’ (191 bp). The efficiency of each primer was tested by running a standard curve in duplicate. The quantification was performed using the ΔΔCT method and the *Rplp0* gene was used as an internal control for normalization. Fold change in mRNA expression was relative to control cells. The specificity of PCR products was confirmed by melting curve analysis. The identity of the amplified products was confirmed by sequencing analysis.

**Bulk RNA-seq**

Total RNA was extracted from Empty and NDRG1-CRISPR cells grown in a 6-well plate using the Trizol (Sigma). RNA was measured with a ND-1000 spectrophotometer (NanoDrop) and its quality assessed with the Bioanalyzer (Agilent Technologies). Indexed libraries were prepared from 800 ng purified RNA with TruSeq Stranded Total RNA Library Prep Gold (Illumina) according to the manufacturer’s instructions. Libraries were quantified using the Bioanalyzer (Agilent Technologies) and Qubit fluorometer (Invitrogen Co.), then pooled such that each index-tagged sample was present in equimolar amounts, with final concentration of the pooled samples of 1.5nM. The pooled samples were subject to cluster generation and sequencing using an Illumina NextSeq 550 System (Illumina) in a 2x75 paired-end format. The raw sequence files generated (.fastq files) underwent quality control analysis using FastQC (http://www.bioinformatics.babraham.ac.uk/projects/fastqc). Adapter was removed with Trimmomatic [Bolger, A. M., Lohse, M., & Usadel, B. (2014). Trimmomatic: A flexible trimmer for Illumina Sequence Data] and reads were aligned with STAR algorithm [14] and read count was performed through RSEM [15]. Read counts were normalized with variance stabilizing transformation prior differential expression analysis with DEseq2 R package [7]. Significantly deregulated genes were analyzed through “ClusterProfiler” R package [16].

**Inverse Matrigel invasion assay**

Inverse Matrigel invasion assay was carried as described in Guerra et al. [17]. Briefly, 100 μl of 50% Matrigel in serum-free medium was pipetted into transwell inserts in a 24-well tissue culture plate. After 30 min incubation at 37°C, the transwell inserts were inverted and 100 μl of cell suspension (10,000 cells/transwell) was pipetted onto the porous membrane, followed by 4h incubation at 37°C to allow cell attachment. The plate was then turned right-side-up and each transwell insert was placed in a well containing serum-free medium. Complete medium was pipetted on top of the set matrigel and incubated for 5 d at 37°C. A staining solution (4 μM Calcein AM) was then added on top of the Matrigel and in the well. After 1h incubation at 37°C, cells were analyzed by confocal microscopy using a 20x objective. Z-sections were assembled using ImageJ/Fiji to obtain volumetric/3D projections, and invasive cells were then counted in the samples.

**Supplemental Figures and Legends**

**
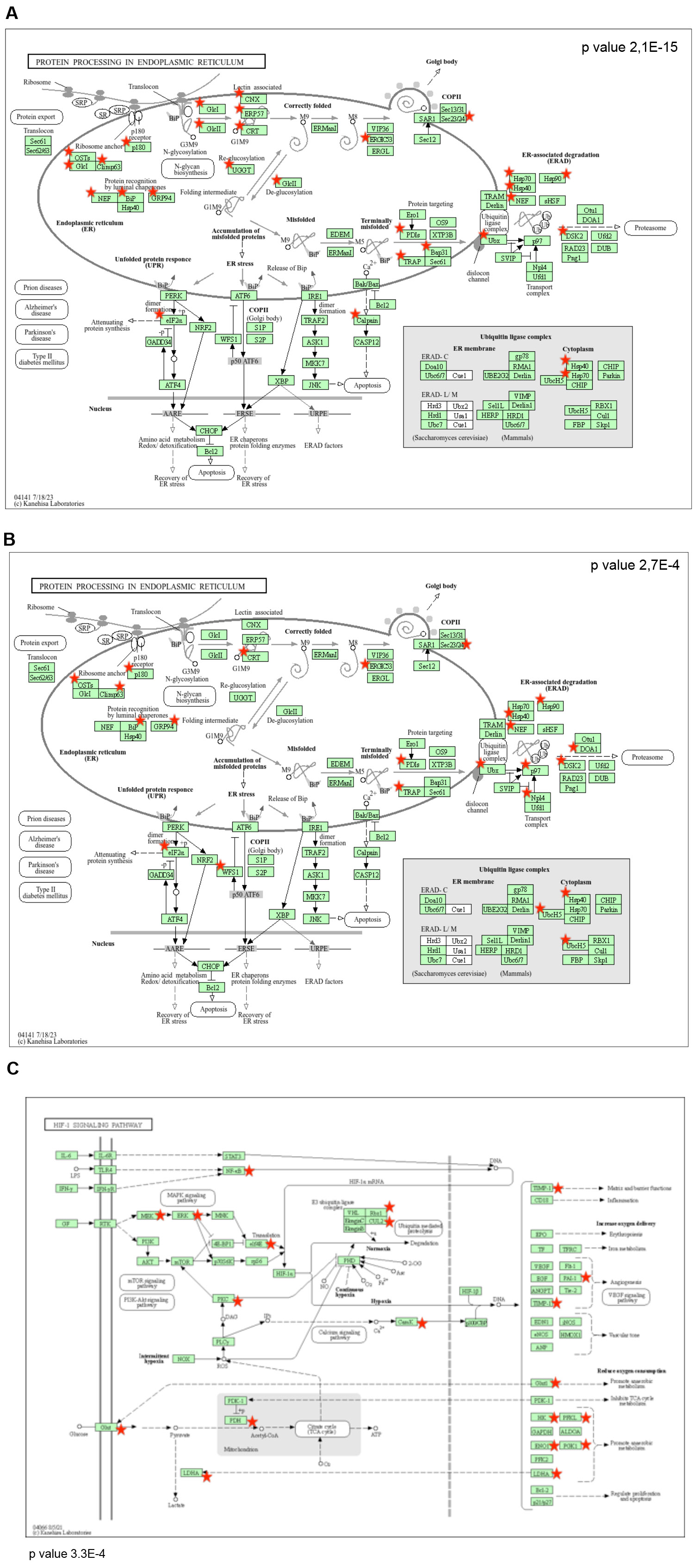
**

**Supplementary Figure 1.** DAVID KEGG pathway analysis. A) Protein processing and endoplasmic reticulum is visualized. Data derived form Supplementary MS/MS Data 1, healthy *vs* tumor samples. B) Protein processing and endoplasmic reticulum is visualized. Data derived form Supplementary MS/MS Data 2, MCF-7 *vs* MDA-MB-231. C) HIF-1 signaling KEGG pathway is visualized. Data derived form Supplementary MS/MS Data 2, MCF-7 *vs* MDA-MB-231. The red stars indicate differentially expressed proteins involved in the pathway and identified by MS/MS.


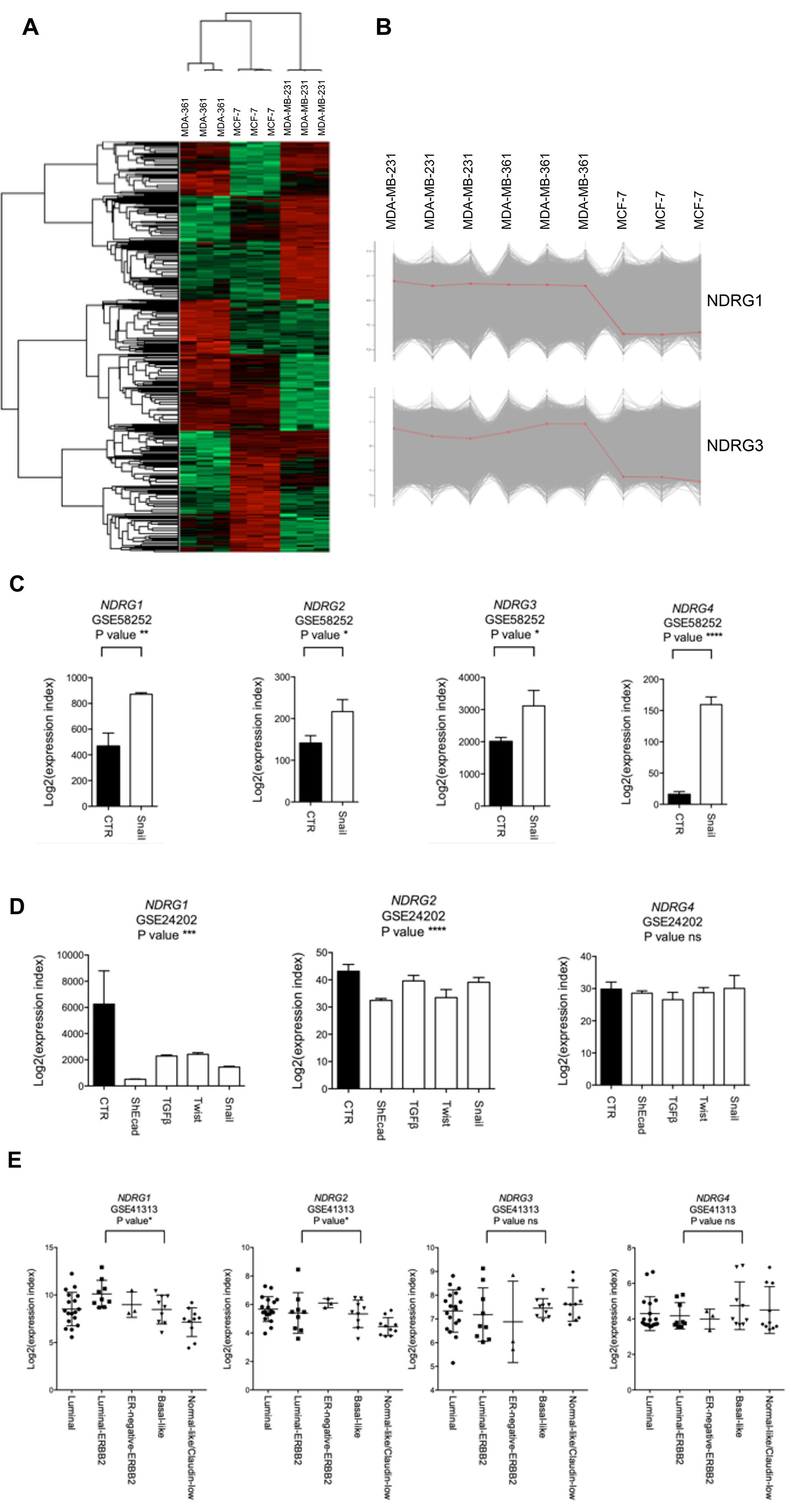


**Supplementary Figure 2**. GEO analysis of BC datasets. A) Heat map based on Euclidean distance showing a significant separation between the MDA-361, MCF-7 and MDA-MB-231 cells. Each row of the heat map represents a protein, and each column represents an independent sample. Three main clusters were identified from the hierarchical clustering, and their pattern is reported. B) The profile plot of NDRG1 and NDRG3 is reported. C) Analysis of *NDRG* family mRNA expression in MCF-7 cells was performed using GEO dataset. Scatter dot plots show *NDRG* family members expression levels in MCF-7 cells from the GEO dataset GSE58252. The p-value for the comparison between the two groups was determined using the Student’s t-test. p-value * < 0.05, ** < 0.01, p-value **** < 0.0001. D) Analysis of mRNA expression in breast cancer cell lines was performed using GEO dataset. Box plots show *NDRG* members expression levels in immortalized HMLE breast epithelial cells retrovirally transduced in culture with vectors encoding EMT-inducing genes. Data were obtained from the GEO dataset GSE24202. The p-value for the comparison between different groups was determined using the ANOVA test. p-value *** < 0.001, **** < 0.0001. E) Analysis of *NDRG* family mRNA expression in breast cancer cells was performed using GEO dataset. Scatter dot plots show *NDRG* family members expression levels in MCF-7 cells from the GEO dataset GSE41313. The p-value for the comparison between different groups was determined using the ANOVA test. p-value * < 0.05.


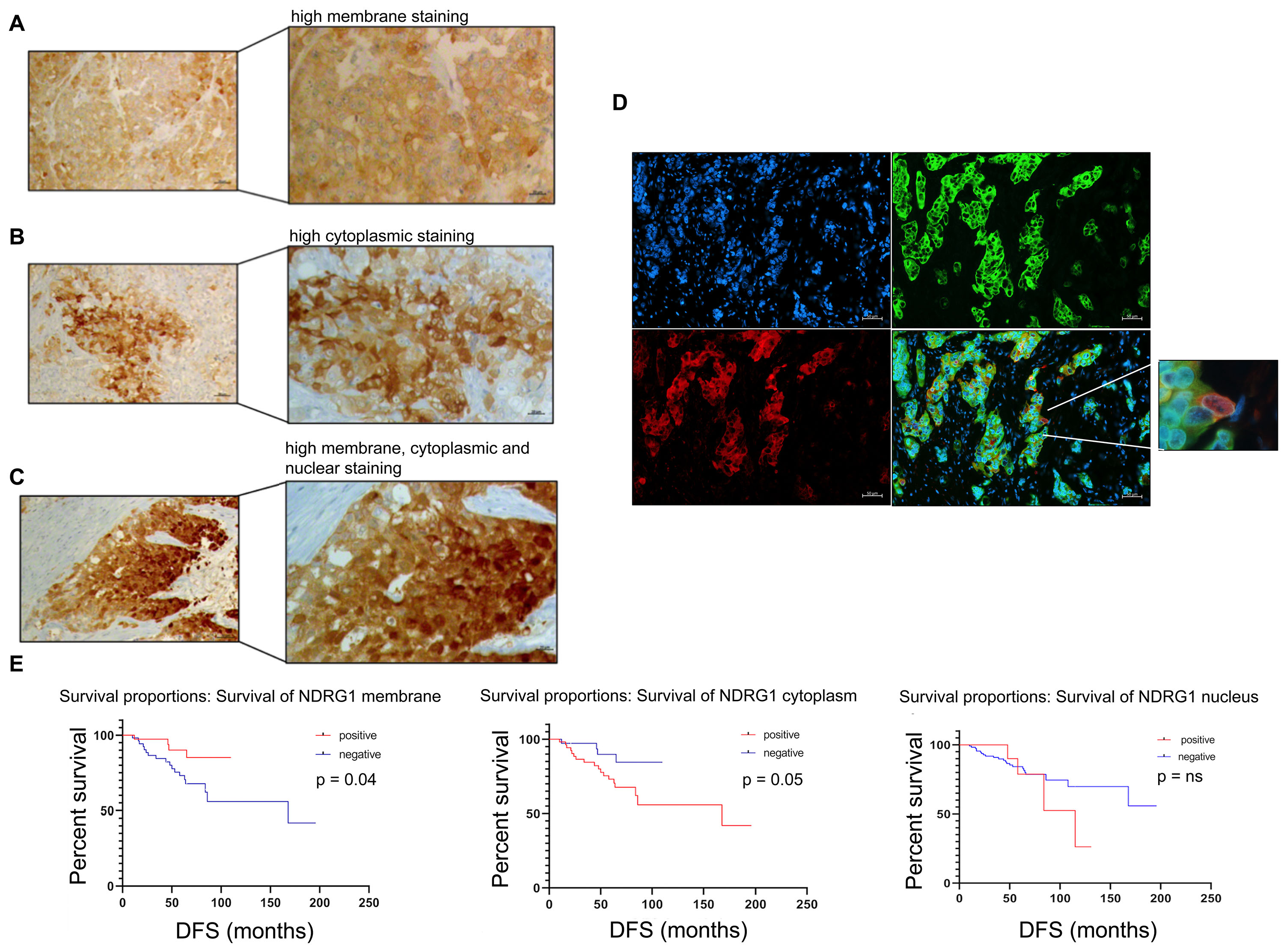


**Supplementary Figure 3.** Immunohistochemical patterns of NDRG1 expression. A) high membrane staining; B) high cytoplasmic staining; C) high membrane, cytoplasmic and nuclear staining. 20x (left) and 40x (right) magnification. Images were obtained on an Axion Image 2 upright microscope (Zeiss, Oberkochen, Germany) with an Axiocam 512 color camera. Scale bar = 20 µm. D) A representative tissue sample stained with NDRG1 and Pan Cytokeratin_488 (green) antibodies and detected with Alexa Fluor 568 (red) secondary antibodies and nucleus in blue (DAPI stained), respectively, prior to fluorescence microscopy analysis. The square includes nuclear staining of NDRG1 (original magnification, ×200). Scale bar=20 µm. Images were obtained on an Axion Image 2 upright microscope (Zeiss, Oberkochen, Germany) with an Axiocam 512 color camera. E) Kaplan–Maier curve analysis. Kaplan–Maier curve by positive versus negative NDRG1 expression in Membrane (p=0.04); Cytoplasm (p=0.05) and Nucleus (p=ns, not significant) of BC patients.


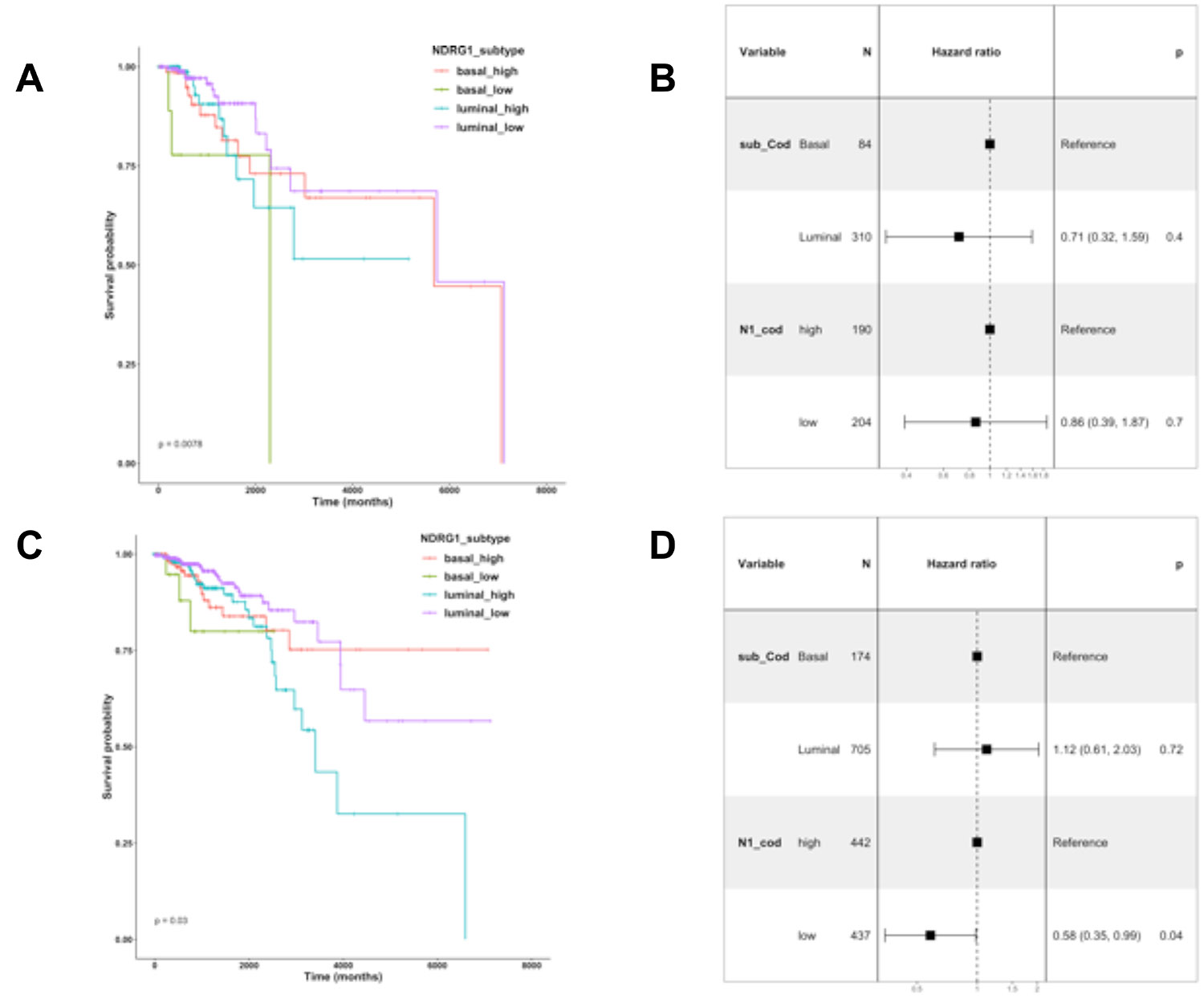


**Supplementary Figure 4.** *NDRG1* prognostic significance in a Breast Cancer cohort. A) Kaplan-Meier plot (left panel) for disease-free survival (DFS) according to *NDRG1* high and low expression derived from a discovery set of 707 luminal and 174 basal cases extracted from transcriptomic profiling of RNAseq. B) Forest plots (right panel) visualize HR and *p*‐value obtained from multivariate analysis of *NDRG1* and subtypes groups. C) Kaplan-Meier plot (left panel) for overall survival (OS) according to *NDRG1* high and low expression derived from a discovery set of 707 luminal and 174 basal cases extracted from transcriptomic profiling of RNAseq. D) Forest plots (right panel) visualize HR and *p*‐value obtained from multivariate analysis of *NDRG1* and subtypes groups.


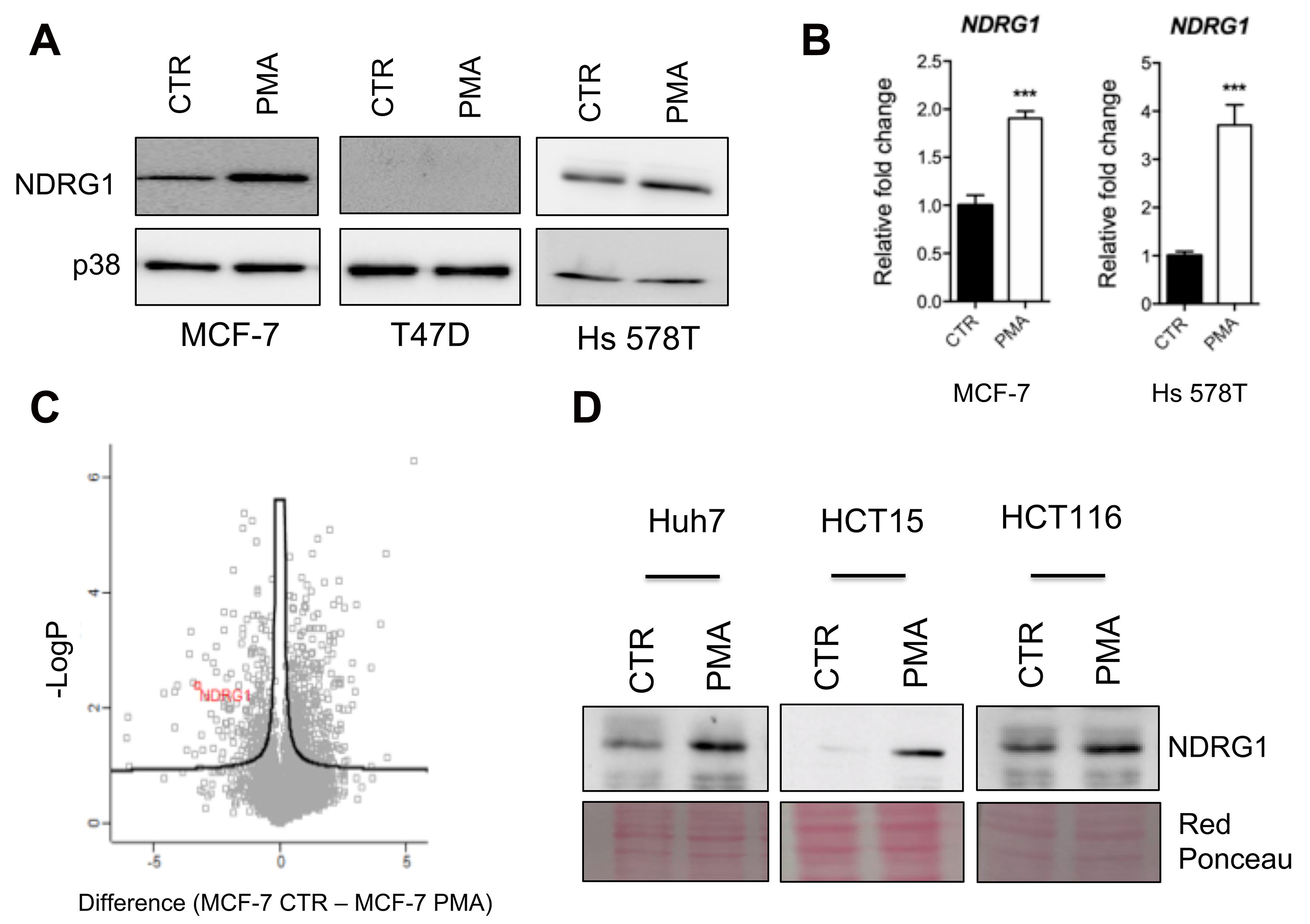


**Supplementary Figure 5.** PMA treatment increases NDRG1 expression in different cell lines. A) Western blotting analysis for NDRG1 of lysates obtained from MCF-7, T47D and Hs 578T cells exposed to PMA (100 nM) for 24 h. B) RT-qPCR in MCF-7 and Hs 578T cells. The p-value was calculated using the Student’s t-test. The error bar represents ± SD. p-value *** < 0.001. C) Volcano plot of published MS/MS data [18] in MCF-7 versus MCF-7 PMA treated cells. NDRG1 is highlighted. D) Western blotting analysis for NDRG1 of lysates obtained from Huh7, HCT-15, and HCT-116 cells exposed to PMA (100 nM) for 24 h.


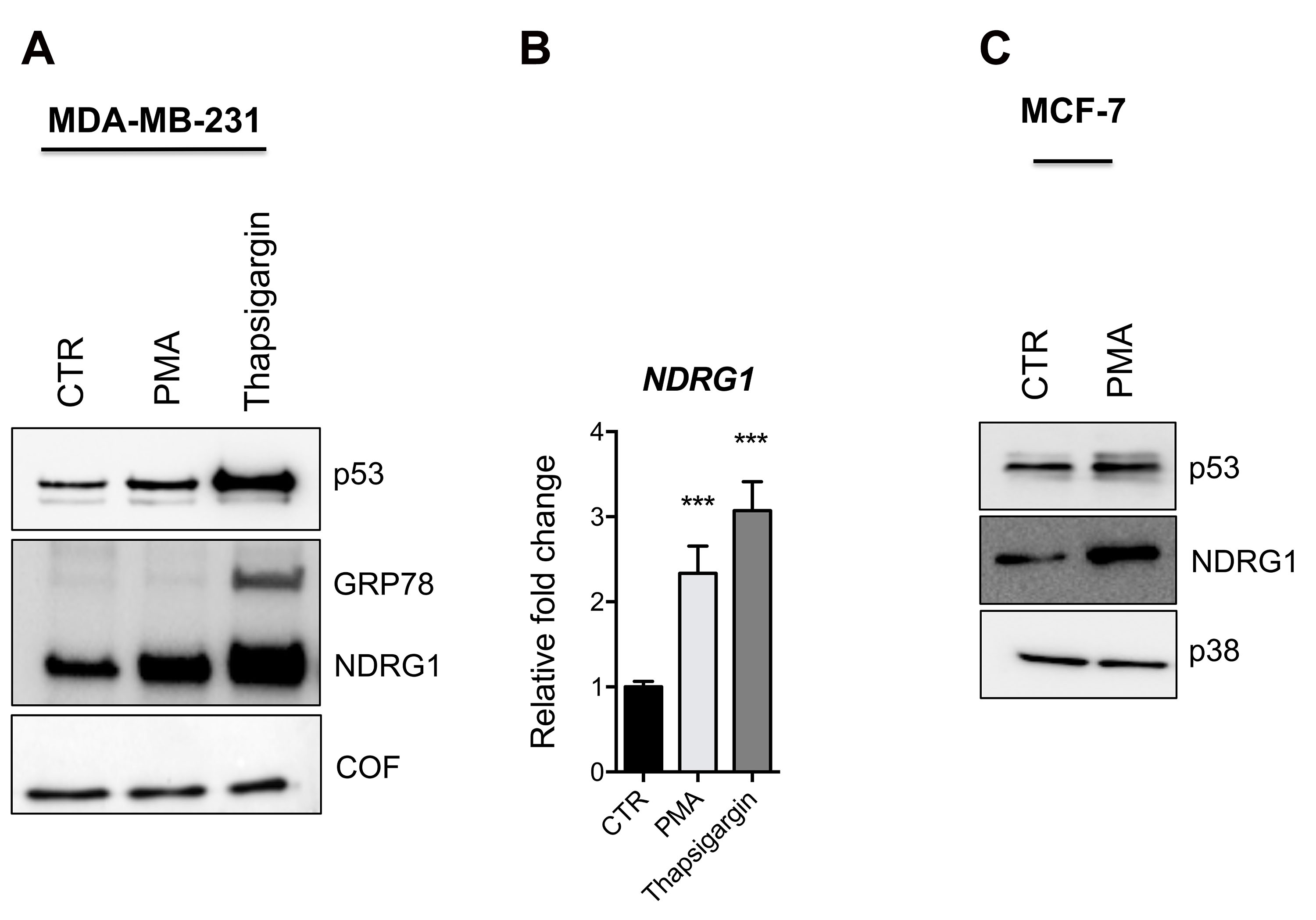


**Supplementary Figure 6.** PMA and thapsygargin increase the expression of p53 in different cell models. A) Western blotting analysis for p53, GRP78, NDRG1 of lysates obtained from MCF-7 cells exposed to PMA (100 nM) and thapsygargin (1 μM) for 24 h. Cofilin was used as a loading control. B) RT-qPCR of *NDRG1* mRNA in control and MDA-MB-231 cells exposed to PMA (100 nM) and thapsygargin (1 μM) for 24 h. The p-value was calculated using the Student’s t-test. The error bar represents ± SD. p-value *** < 0.001. C) Western blotting analysis for p53, and NDRG1 of lysates obtained from MCF-7 cells exposed to PMA (100 nM) for 24 h. p38 was used as a loading control.


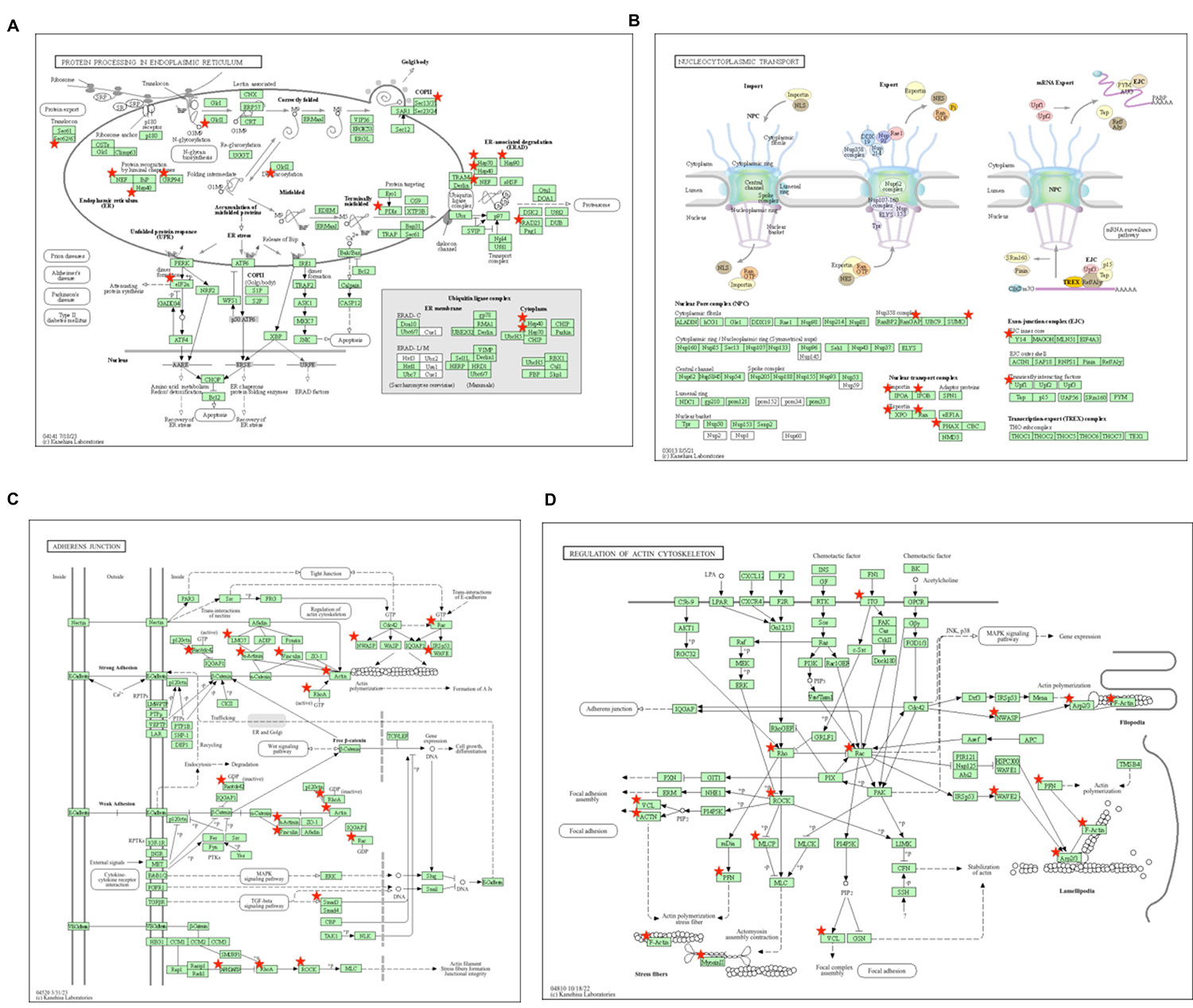


S**upplementary Figure 7.** KEGG pathway are visualized. Pathway maps of A) Protein processing in endoplasmic reticulum, B) Nucleocytoplasmatic transport, C) Adherens Junction. Data derived form Supplementary MS/MS Data 4, Empty *vs* NDRG1-CRISPR. The red stars indicate differentially expressed proteins involved in the pathway and identified by MS/MS.

S**upplementary Figure 8.** Expression of genes involved in the significantly enriched pathway “Extracellular matrix organization”.

**Supplemental References**

1. Wolff AC, Hammond MEH, Allison KH, Harvey BE, Mangu PB, Bartlett JMS, Bilous M, Ellis IO, Fitzgibbons P, Hanna W, Jenkins RB, Press MF, Spears PA, Vance GH, Viale G, McShane LM, Dowsett M (2018) Human [Epidermal Growth Factor Receptor 2 Testing in Breast Cancer: American Society of Clinical Oncology/College of American Pathologists Clinical Practice Guideline Focused Update.](https://pubmed.ncbi.nlm.nih.gov/29846122/) J Clin Oncol 36(20):2105-2122. https://doi.org/10.1200/JCO.2018.77.8738

2. Villodre ES, Gong Y, Hu X, Huo L, Yoon EC, Ueno NT, Woodward WA, Tripathy D, Song J, Debeb BG (2020) NDRG1 Expression Is an Independent Prognostic Factor in Inflammatory Breast Cancer. Cancers (Basel) 12(12):3711. https://doi.org/10.3390/cancers12123711

3. Sgroi DC, Treuner K, Zhang Y, Piper T, Salunga R, Ahmed I, Doos L, Thornber S, Taylor KJ, Brachtel E, Pirrie S, Schnabel CA, Rea D, Bartlett JMS (2022) Correlative studies of the Breast Cancer Index (HOXB13/IL17BR) and ER, PR, AR, AR/ER ratio and Ki67 for prediction of extended endocrine therapy benefit: a Trans-aTTom study. Breast Cancer Res 24(1):90. https://doi.org/10.1186/s13058-022-01589-x

4. Salgado R, Denkert C, Demaria S, Sirtaine N, Klauschen F, Pruneri G, Wienert S, Van den Eynden G, Baehner FL, Penault-Llorca F, Perez EA, Thompson EA, Symmans WF, Richardson AL, Brock J, Criscitiello C, Bailey H, Ignatiadis M, Floris G, Sparano J, Kos Z, Nielsen T, Rimm DL, Allison KH, Reis-Filho JS, Loibl S, Sotiriou C, Viale G, Badve S, Adams S, Willard-Gallo K, Loi S; International TILs Working Group 2014 (2015) The evaluation of tumor-infiltrating lymphocytes (TILs) in breast cancer: recommendations by an International TILs Working Group 2014. Ann Oncol 26(2):259-71. https://doi.org/10.1093/annonc/mdu450

5. Mangia A, Saponaro C, Vagheggini A, Opinto G, Centonze M, Vicenti C, Popescu O, Pastena M, Giotta F, Silvestris N (2019) Should Tumor Infiltrating Lymphocytes, Androgen Receptor, and FOXA1 Expression Predict the Clinical Outcome in Triple Negative Breast Cancer Patients? Cancers (Basel) 11(9):1393. https://doi.org/10.3390/cancers11091393

6. Bray NL, Pimentel H, Melsted P, Pachter L (2016) Near-optimal probabilistic RNA-seq quantification. Nat Biotechnol 34(5):525-7. https://doi.org/10.1038/nbt.3519

7. Love MI, Huber W, Anders S (2014) Moderated estimation of fold change and dispersion for RNA-seq data with DESeq2. Genome Biol 15(12):550. https://doi.org/10.1186/s13059-014-0550-8

8. Colaprico A, Silva TC, Olsen C, Garofano L, Cava C, Garolini D, Sabedot TS, Malta TM, Pagnotta SM, Castiglioni I, Ceccarelli M, Bontempi G, Noushmehr H (2016) TCGAbiolinks: an R/Bioconductor package for integrative analysis of TCGA data. Nucleic Acids Res 44(8):e71. https://doi.org/10.1093/nar/gkv1507

9. Cox J, Mann M (2008) Max Quant enables high peptide identification rates, individualized p.p.b.-range mass accuracies and proteome-wide protein quantification. Nat Biotechnol 26(12):1367-1372. https://doi.org/10.1038/nbt.1511

10. Tyanova S., Temu T., Sinitcyn P., Carlson A., Hein M.Y., Geiger T., Mann M., Cox J (2016) The Perseus computational platform for comprehensive analysis of (prote)omics data. Nat. Methods 13: 731-740. https://doi.org/ 10.1038/nmeth.3901

11. Cox J, Hein MY, Luber CA, Paron I, Nagaraj N, Mann M (2014) Accurate proteome-wide label-free quantification by delayed normalization and maximal peptide ratio extraction, termed MaxLFQ. Mol Cell Proteomics 13(9):2513-2526. https://doi.org/10.1074/mcp.M113.031591

12. Deutsch EW, Bandeira N, Sharma V, Perez-Riverol Y, Carver JJ, Kundu DJ, García-Seisdedos D, Jarnuczak AF, Hewapathirana S, Pullman BS, Wertz J, Sun Z, Kawano S, Okuda S, Watanabe Y, Hermjakob H, MacLean B, MacCoss MJ, Zhu Y, Ishihama Y, Vizcaíno JA (2020) The ProteomeXchange consortium in 2020: enabling 'big data' approaches in proteomics. Nucleic Acids Res 48(D1):D1145-D1152. https://doi.org/10.1093/nar/gkz984

13. Perez-Riverol Y, Csordas A, Bai J, Bernal-Llinares M, Hewapathirana S, Kundu DJ, Inuganti A, Griss J, Mayer G, Eisenacher M, Pérez E, Uszkoreit J, Pfeuffer J, Sachsenberg T, Yilmaz S, Tiwary S, Cox J, Audain E, Walzer M, Jarnuczak AF, Ternent T, Brazma A, Vizcaíno JA (2019) The PRIDE database and related tools and resources in 2019: improving support for quantification data. Nucleic Acids Res 47(D1):D442-D450. https://doi.org/10.1093/nar/gky1106

14. Dobin A, Davis CA, Schlesinger F, Drenkow J, Zaleski C, Jha S, Batut P, Chaisson M, Gingeras TR (2013) STAR: ultrafast universal RNA-seq aligner. Bioinformatics 29(1):15-21. doi: 10.1093/bioinformatics/bts635

15. Li B, Dewey CN (2011) RSEM: accurate transcript quantification from RNA-Seq data with or without a reference genome. BMC Bioinformatics 12:323. doi: 10.1186/1471-2105-12-323

16. Wu T, Hu E, Xu S, Chen M, Guo P, Dai Z, Feng T, Zhou L, Tang W, Zhan L, Fu X, Liu S, Bo X, Yu G (2021) clusterProfiler 4.0: A universal enrichment tool for interpreting omics data. Innovation (Camb) 2(3):100141. doi: 10.1016/j.xinn.2021.100141

17. Guerra E, Relli V, Ceci M, Tripaldi R, Simeone P, Aloisi AL, Pantalone L, La Sorda R, Lattanzio R, Sacchetti A, Havas K, Guarnieri S, Vergara D, Fournier I, Salzet M, Tinari N, Piantelli M, Trerotola M, Alberti S (2022) Trop-2, Na+/K+ ATPase, CD9, PKCα, cofilin assemble a membrane signaling super-complex that drives colorectal cancer growth and invasion. Oncogene 41(12):1795-1808. https://doi.org/10.1038/s41388-022-02220-1

18. Vergara D, Ravaioli S, Fonzi E, Adamo L, Damato M, Bravaccini S, Pirini F, Gaballo A, Barbano R, Pasculli B, Franck J, Fournier I, Salzet M, Maffia M (2020) Carbonic Anhydrase XII Expression Is Modulated during Epithelial Mesenchymal Transition and Regulated through Protein Kinase C Signaling. Int J Mol Sci 21(3):715. https://doi.org/ 10.3390/ijms21030715.
